# Supplementary material for: Spatial interpolation of health and demographic variables: Predicting malaria indicators with and without covariates
Source: PLoS One. 2025 May 29;20(5):e0322819. doi: 10.1371/journal.pone.0322819 (PMC12121779; doi:10.1371/journal.pone.0322819)
Supplement: S1 Text — (DOCX) [file pone.0322819.s001.docx]

# DHS indicators

## Proportion of Fula

This indicator shows the proportion of the population (men and women) belonging to the Fula ethnic group. Fula, Fulani or Pulaar have been shown to be less susceptible to malaria due to higher antibody levels and other protective effects (type O blood, etc.) [1]. In addition, the nomadic lifestyle of some Fula ethnic groups in Senegal may also influence malaria prevention behaviour.

## Stunting in children

Stunting is a measure of chronic (i.e. repeated) nutritional deficiency. For children, it is measured based on the children height for a specific age and sex. This indicator is defined as the proportion of children under five who are moderately or severely stunted, i.e. children whose height-for-age is less than two standard deviations from the median defined by the Child Growth Standards of the World Health Organisation (WHO) [2–4].

## Anemia prevalence in children

Anemia refers to the lack of haemoglobin in blood, and it is measured by DHS using prick blood tests. It is defined as the proportion of children aged six months to five years with mild, moderate or severe anemia, i.e. with less than 11 grams of haemoglobin per blood decilitre [2,3]. Note that children under six months of age are not included in this indicator as children have higher levels of haemoglobin at birth and up to six months of age, which may lead to overestimates of anemia prevalence [3]. Anemia is often associated with malnutrition and malaria infection.

## Access to basic sanitation service

This indicator is defined as the proportion of households having access to a basic sanitation service. According to the WHO/UNICEF Joint Monitoring Programme classification standards for Water Supply and Sanitation, basic sanitation services are characterised as the use of improved sanitation facilities that are not shared with any other households [5]. Note that improved sanitation facilities are toilet facilities that hygienically separate excreta from human contact (i.e. flush to piped sewer system/septic tank/pit latrine, ventilated improved pit latrine, pit latrine with slab and composting toilet) [2,3].

## Wealth index

The DHS wealth index is a composite indicator of household ownership of a range of assets and services, such as ownership of a television, car or land, the type of drinking water source and sanitation facilities, and dwelling characteristics such as flooring materials and number of people sleeping per room, among others. Using Principal Component Analysis, households are assigned scores according to their assets and services, accounting for urban/rural specificities, with higher scores meaning wealthier households. At the national scale, urban and rural scores are combined into a single standardised score with a mean of 0 and a standard deviation of 1 [3]. In this study, following [6], we used this combined score, averaged at the cluster level, as the household wealth index.

## Literacy rate in women

The literacy rate in women is the proportion of women aged 15 to 49 who are literate. In DHS, literacy is assessed by the interviewer by asking the respondent to read a sentence or part of a sentence in English or in the respondent native language. In this study, we defined literate women as those with more than secondary school education or women who can read at least part of a sentence, following [3,4].

## Insecticide-treated net coverage

Insecticide-treated Nets (ITN) include all types of mosquito nets, including those that need to be treated/retreated and those that are factory treated with an efficacy of about three years (known as long-lasting insecticidal nets (LLIN)), which are the most recommended [7]. Here we use ITN to refer to LLIN only. In this study, we used the following indicators of ITN coverage, as recommended by the Roll Back Malaria [8] and following previous work [9,10]: (1) the proportion of households with at least one ITN, (2) the proportion of households with at least one ITN for every two people that slept at the house the night before the survey, and (3) the proportion of population with access to an ITN in their household (i.e. proportion of household population who slept under an ITN if each ITN in the household was used by up to two people).

## Indoor residual spraying coverage

Following the Roll Back Malaria [8] and previous work [11,12], we calculated the proportion of households sprayed with residual insecticide in the 12 months prior to the survey.

# Geospatial covariates

All geospatial covariates are listed in Table 1 and further detailed in the following sections.

**Table 1.** Geospatial covariates and their characteristics

| **Dataset** | **Variables** | **Date** | **Spatial resolution (m)** | **Type** | **Source** |
| --- | --- | --- | --- | --- | --- |
| CHELSA | Precipitation, temperature at 2 m, potential evapotranspiration, near-surface relative humidity, CMI | 1988-2018 | 1000 | Continuous | CHELSA (https://chelsa-climate.org/) |
| MODIS LST | Day LST, night LST, daily LST range | 2015-2017 | 1000 | Continuous | MODIS (http://modis.gsfc.nasa.gov/) |
| Vegetation indices | NDVI, NDWI, NDMI | 2015-2017 | 10 | Continuous | Computed from Sentinel-2 L1C composites of the JRC (https://forobs.jrc.ec.europa.eu/sentinel/sentinel2_composite) |
| WSF | Settlements | 2015 | 10 | Categorical | German Aerospace Center (https://geoservice.dlr.de/) |
| GHSL | Residential built-up surface | 2018 | 10 | Continuous | JRC (https://ghsl.jrc.ec.europa.eu/download.php) |
|  | Building height |  | 100 |  |  |
| Dynamic World | Water, trees, flooded vegetation, crops, grass, bare ground, shrubland | 2017 | 10 | Categorical | Google and the World Resources Institute (https://dynamicworld.app/) |
| VIIRS nighttime lights | Average and median nighttime lights | 2017 | 500 | Continuous | Earth Observation Group (https://eogdata.mines.edu/products/vnl/#annual_v2) |
| Distances to OSM features | Distance to major roads, waterways, education facilities and health facilities | 2023 | 100 | Continuous | OSM (www.openstreetmap.org) |
| Accessibility maps | Travel time to major cities | 2015 | 1000 | Continuous | MAP (https://malariaatlas.org/project-resources/accessibility-to-healthcare/) |
|  | Walking-only and motorized travel time to healthcare facilities | 2019 |  |  |  |
| WorldPop products | Population counts | 2017 | 100 | Continuous | WorldPop Project (https://www.worldpop.org/) |
|  | Births, pregnancies | 2015 | 1000 |  |  |
| SIDE | Proportion of Wolof, Fula, Serer, Diola, Mandingue, Soninke, not Senegalese and other ethnic groups | 2013 | 1000 | Continuous | ETH Zurich (https://icr.ethz.ch/data/side/) |
| SRTM | Elevation | 2000 | 30 | Continuous | US Geological Survey (USGS) (http://eros.usgs.gov/elevation-products) |
| GLW | Density of goat, cattle, pig, poultry, sheep | 2010 | 1000 | Continuous | FAO in collaboration with ILRI, the University of Oxford and the Université Libre de Bruxelles (http://www.fao.org) |

## CHELSA

We calculated the mean year-monthly precipitation, temperature at 2 m, potential evapotranspiration, near-surface relative humidity and climate moisture index (CMI) over period 1988-2018 from CHELSA (Climatologies at high resolution for the earth’s land surface areas) v2.1 climate data [13]. CHELSA are statistically downscaled model output estimates at 30 arcsec (~1 km). While showing similar accuracy to WorldClim temperature estimates, previous work shows that CHELSA precipitation data are more accurate [13] and should be preferred to interpolated data (i.e. WorldClim) in areas where weather stations are sparsely distributed [13,14]. For replicability of this work beyond Senegal, we used CHELSA climate data over other existing products.

## MODIS LST

From MODIS Aqua and Terra data collections (MYD11A1 v006 and MOD11A1 v006) [15,16], we extracted the mean year-monthly day and night land surface temperature (LST) and the mean daily LST range (the difference between the maximum and minimum daily LST) at 1 km resolution over 2015-2017.

## Vegetation indices

Normalized difference vegetation, water and moisture indices (NDVI, NDWI, NDMI) were computed from the 2015-2017 Sentinel-2 L1C cloud-free composites (10 m resolution) of the Joint Research Centre (JRC) [17]. The methodology to create these Sentinel-2 L1C composites is tailored to the pan-tropical zone (e.g. Senegal) where the large cloud cover sometimes leads to the lack of cloud-free images for months [17]. Although not atmospherically corrected, these composites can be used directly for extracting vegetation indices, as done in [18], as these are less influenced by atmospheric effects [17,18]. So far, existing Sentinel-2 L2A composites (e.g. the Sentinel-2 Global Mosaic service) are often prone to errors due to the quality of the scene classification of Sentinel-2 L2A data and cloud masks provided by the Copernicus service [17].

## World Settlement Footprint

The World Settlement Footprint (WSF) is a global binary mask of human settlements available at 10 m spatial resolution [19]. This dataset was created by the German Aerospace Center from Sentinel-1 radar images and Landsat-8 optical images. Here we used the WSF for the year 2015.

## Global Human Settlement Layer

As human settlement data, we used the 2018 Global Human Settlement Layer (GHSL), which represents built-up areas (in square metres) for residential areas (GHS-BUILT-S (RES)). These data are derived from Sentinel-2 L1C cloud-free composites at 10 m spatial resolution [20]. In addition, we used the averaged building height (GHS-BUILT-H) from the same project, which is available at 100 m spatial resolution and is derived from digital elevation models (AW3D30, 2006-2011 and SRTM30, 2000) and Sentinel-2 cloud-free composites [21].

## Dynamic World

Land use land cover (LULC) data were extracted from the Dynamic World global LULC dataset available in near-real time at 10 m spatial resolution. This dataset is derived from Sentinel-2 satellite imagery using deep learning algorithms. It provides the classification probability of nine LULC categories: water, trees, flooded vegetation, crops, grass, built area, bare ground, shrubland, and snow [22]. In this study, the snow category was discarded, as well as the built area, due to overlap with other covariates (i.e. GHSL and WSF). All LULC data were aggregated over 2017 using the most frequent LULC class on average. Classification validation shows an overall agreement of 73.8 % with experts, outperforming current global LULC products such as ESA CCI 2018 and ESA CGLS ProbaV 2019 [22].

## VIIRS nighttime lights

The median and the average VIIRS nighttime lights over 2017 were obtained from the Earth Observation Group (Annual VNL v2.1) [23]. They are based on monthly cloud-free radiance averages made from NASA/NOAA VIIRS data. Compared to other existing nightlights products such as DMSP-OLS, VIIRS nighttime lights offer several improvements, including a higher radiometric and spatial resolution (500 m) [24].

## Distances to OpenStreetMap features

Distances (100 m resolution) to different types of OpenStreetMap (OSM) features were calculated, such as the distance to major roads (i.e. motorways, trunks, primary, secondary, and tertiary roads), waterways, education (i.e. kindergarten, schools, classes, college, and university) and health facilities, using the most recent OSM data available at the time of the study.

## Accessibility maps

The Malaria Atlas Project (MAP) provides an accessibility map for 2015 in terms of travel time to the nearest city, defined as a contiguous area with at least 1,500 inhabitants per square kilometre or a majority of built-up land cover coincident with a population centre of at least 50,000 inhabitants [25]. Additionally, they also provide maps of the travel time to healthcare facilities (2019), with and without access (i.e. walking-only) to motorized transport [26]. These datasets are based on friction surfaces; maps of the overall speed required to cross any pixel, given several factors that influence the cost of moving through the landscape, i.e. roads, water channels, topography, landcover and national borders among others. These accessibility datasets are all available at 1 km spatial resolution.

## WorldPop products

Gridded data on population counts were acquired from the WorldPop project for Senegal in 2017. These grids available at 100 m spatial resolution are derived from the disaggregation of census data using Random Forest-based dasymetric redistribution with various geospatial covariates (e.g. land use and land cover, nighttime lights, etc.) [27]. In addition, we also used gridded data on births and pregnancies available at 1 km spatial resolution for 2015 [28].

## Spatially Interpolated Data on Ethnicity

Spatially Interpolated Data on Ethnicity (SIDE) is a collection of 1 km spatial resolution gridded maps of ethnic and religious settlement patterns in 47 low- and middle-income countries [29]. This dataset constitutes an improvement over the GeoEPR vector data, as it shows variations in ethnic settlements at a finer scale within regions [29]. SIDE maps are based on spatial interpolation of ethnic/religious composition data from the Demographic and Health Surveys. Grid cells contain the proportion of people belonging to each ethnic or religious group for a specific country. Here, we used gridded maps of the different ethnic groups in Senegal for 2013, i.e. Wolof, Fula, Serer, Diola, Mandingue, Soninke, not Senegalese and other ethnic groups.

## SRTM

A Digital Elevation Model at 30 m spatial resolution was obtained from the NASA Shuttle Radar Topography Mission (SRTM) [30].

## Gridded Livestock of the World

We acquired gridded maps on the distribution of goats, cattle, pigs, poultry, and sheep at 1 km spatial resolution for 2010 from the Gridded Livestock of the World (v2.01) project (GLW). These maps are based on the disaggregation of sub-national census data using machine learning and environmental factors in similar agro-ecological zones [31].

# References

1. Seck MC, Thwing J, Fall FB, Gomis JF, Deme A, Ndiaye YD, et al. Malaria prevalence, prevention and treatment seeking practices among nomadic pastoralists in northern Senegal. Malar J. 2017 Dec;16(1):413.

2. Gething P, Tatem A, Bird T, Burgert-Brucker CR. Creating Spatial Interpolation Surfaces with DHS Data. Rockville, Maryland, USA: ICF International; 2015. (DHS Spatial Analysis Reports No. 11).

3. Croft TN, Marshall AMJ, Allen CK. Guide to DHS Statistics 7. Rockville, Maryland, USA: ICF International; 2018.

4. Bosco C, Alegana V, Bird T, Pezzulo C, Bengtsson L, Sorichetta A, et al. Exploring the high-resolution mapping of gender-disaggregated development indicators. J R Soc Interface. 2017 Apr 30;14(129):20160825.

5. World Health Organization (WHO), United Nations Children’s Fund (UNICEF). Progress on drinking water, sanitation and hygiene: 2017 update and SDG baselines. Geneva: WHO and UNICEF; 2017.

6. Georganos S, Gadiaga AN, Linard C, Grippa T, Vanhuysse S, Mboga N, et al. Modelling the Wealth Index of Demographic and Health Surveys within Cities Using Very High-Resolution Remotely Sensed Information. Remote Sens. 2019 Oct 29;11(21):2543.

7. World Health Organization. WHO guidelines for malaria, 30 November 2024. Geneva: World Health Organization; 2024 Nov. https://doi.org/10.2471/B09146.

8. MEASURE Evaluation, The Demographic and Health Surveys Program, President’s Malaria Initiative, Roll Back Malaria Partnership, United Nations Children’s Fund, World Health Organization,. Household Survey Indicators for Malaria Control. 2018.

9. Adigun AB, Gajere EN, Oresanya O, Vounatsou P. Malaria risk in Nigeria: Bayesian geostatistical modelling of 2010 malaria indicator survey data. Malar J. 2015 Apr 14;14(1):156.

10. Ssempiira J, Nambuusi B, Kissa J, Agaba B, Makumbi F, Kasasa S, et al. Geostatistical modelling of malaria indicator survey data to assess the effects of interventions on the geographical distribution of malaria prevalence in children less than 5 years in Uganda. PLOS ONE. 2017 Apr 4;12(4):e0174948.

11. Riedel N, Vounatsou P, Miller JM, Gosoniu L, Chizema-Kawesha E, Mukonka V, et al. Geographical patterns and predictors of malaria risk in Zambia: Bayesian geostatistical modelling of the 2006 Zambia national malaria indicator survey (ZMIS). Malar J. 2010 Feb 1;9(1):37.

12. Giardina F, Kasasa S, Sié A, Utzinger J, Tanner M, Vounatsou P. Effects of vector-control interventions on changes in risk of malaria parasitaemia in sub-Saharan Africa: a spatial and temporal analysis. The Lancet Global Health. 2014 Oct 1;2(10):e601–15.

13. Karger DN, Conrad O, Böhner J, Kawohl T, Kreft H, Soria-Auza RW, et al. Climatologies at high resolution for the earth’s land surface areas. Sci Data. 2017 Sep 5;4(1):170122.

14. Rusk J, Maharjan A, Tiwari P, Chen THK, Shneiderman S, Turin M, et al. Multi-hazard susceptibility and exposure assessment of the Hindu Kush Himalaya. Science of The Total Environment. 2022 Jan 15;804:150039.

15. Wan Z, Hook S, Hulley, G. MODIS/Terra Land Surface Temperature/Emissivity Daily L3 Global 1km SIN Grid V061 [dataset]. NASA EOSDIS Land Processes DAAC; 2021. https://doi.org/10.5067/MODIS/MOD11A1.061.

16. Wan Z, Hook S, Hulley, G. MODIS/Aqua Land Surface Temperature/Emissivity Daily L3 Global 1km SIN Grid V061 [dataset]. NASA EOSDIS Land Processes DAAC; 2021. https://doi.org/10.5067/MODIS/MYD11A1.061.

17. Simonetti D, Pimple U, Langner A, Marelli A. Pan-tropical Sentinel-2 cloud-free annual composite datasets. Data in Brief. 2021 Dec;39:107488.

18. Verhegghen A, Kuzelova K, Syrris V, Eva H, Achard F. Mapping Canopy Cover in African Dry Forests from the Combined Use of Sentinel-1 and Sentinel-2 Data: Application to Tanzania for the Year 2018. Remote Sensing. 2022 Jan;14(6):1522.

19. Marconcini M, Metz-Marconcini A, Üreyen S, Palacios-Lopez D, Hanke W, Bachofer F, et al. Outlining where humans live, the World Settlement Footprint 2015. Sci Data. 2020 Jul 20;7(1):242.

20. Pesaresi M, Huadong G, Blaes X, Ehrlich D, Ferri S, Gueguen L, et al. A Global Human Settlement Layer From Optical HR/VHR RS Data: Concept and First Results. 2013 Oct;

21. Pesaresi M, Politis P. GHS-BUILT-H R2023A - GHS building height, derived from AW3D30, SRTM30, and Sentinel2 composite (2018) [dataset]. European Commission, Joint Research Centre (JRC); 2023 May. https://doi.org/10.2905/85005901-3A49-48DD-9D19-6261354F56FE.

22. Brown CF, Brumby SP, Guzder-Williams B, Birch T, Hyde SB, Mazzariello J, et al. Dynamic World, Near real-time global 10 m land use land cover mapping. Sci Data. 2022 Jun 9;9(1):251.

23. Elvidge CD, Zhizhin M, Ghosh T, Hsu FC, Taneja J. Annual Time Series of Global VIIRS Nighttime Lights Derived from Monthly Averages: 2012 to 2019. Remote Sensing. 2021 Jan;13(5):922.

24. Elvidge CD, Baugh KE, Zhizhin M, Hsu FC. Why VIIRS data are superior to DMSP for mapping nighttime lights. In: Proceedings of the Asia-Pacific Advanced Network [Internet]. 2013. p. 62.

25. Weiss DJ, Nelson A, Gibson HS, Temperley W, Peedell S, Lieber A, et al. A global map of travel time to cities to assess inequalities in accessibility in 2015. Nature. 2018 Jan;553(7688):333–6.

26. Weiss DJ, Nelson A, Vargas-Ruiz CA, Gligorić K, Bavadekar S, Gabrilovich E, et al. Global maps of travel time to healthcare facilities. Nat Med. 2020 Dec;26(12):1835–8.

27. Lloyd CT, Chamberlain H, Kerr D, Yetman G, Pistolesi L, Stevens FR, et al. Global spatio-temporally harmonised datasets for producing high-resolution gridded population distribution datasets. Big Earth Data. 2019 Apr 3;3(2):108–39.

28. Tatem AJ, Campbell J, Guerra-Arias M, de Bernis L, Moran A, Matthews Z. Mapping for maternal and newborn health: the distributions of women of childbearing age, pregnancies and births. Int J Health Geogr. 2014 Jan 4;13(1):2.

29. Müller-Crepon C, Hunziker P. New spatial data on ethnicity: Introducing SIDE. Journal of Peace Research. 2018 Sep 1;55(5):687–98.

30. Farr TG, Rosen PA, Caro E, Crippen R, Duren R, Hensley S, et al. The Shuttle Radar Topography Mission. Reviews of Geophysics. 2007;45(2):RG2004.

31. Robinson TP, Wint GRW, Conchedda G, Boeckel TPV, Ercoli V, Palamara E, et al. Mapping the Global Distribution of Livestock. PLOS ONE. 2014 mai;9(5):e96084.
