# Supplementary material for: Spatial interpolation of health and demographic variables: Predicting malaria indicators with and without covariates
Source: PLoS One. 2025 May 29;20(5):e0322819. doi: 10.1371/journal.pone.0322819 (PMC12121779; doi:10.1371/journal.pone.0322819)
Supplement: S2 Text — (DOCX) [file pone.0322819.s002.docx]

# Modelling approaches

## Kriging

Kriging estimates the value ($\hat{z}_{0}$) of the random variable $z$ at location $s_{0}$ as the weighted average of its value ($z_{i}$) at the $n$ sample points at location $s_{i}$ [1], such as inverse distance weighting (IDW). The main difference with IDW lies in the estimation of the interpolation weights; in ordinary and universal kriging, these are calculated based on a semi-variogram function that recovers the spatial autocorrelation pattern in the data. The interpolation weights are estimated in the following steps.

1. Building the empirical variogram

The variogram function $\gamma\left( h \right)$expresses the average dissimilarity, i.e. the semi-variance, of the values of the random variable z at different sample points as a function of the distance $h$separating these sample points:

|  | $\gamma(h)=\frac{1}{2}{(z\left( s+h \right)-z\left( s \right))}^{2}$ | (1) |
| --- | --- | --- |

The empirical variogram is built by fitting (1) between all pairs of sample points in the dataset and averaging the resulting semi-variance using distance intervals (called variogram width). In this paper, the variogram width and the cutoff (i.e. the maximum distance considered in the empirical variogram) were tuned using a 50-repeated 4-fold random cross-validation. For the cutoff value, values from 100 km to the maximum distance between observation points in the dataset were tested. The variogram width values were based on the cutoff values, using the cutoff value divided by 15 [2]. The empirical variogram was constructed under the assumption of anisotropy, i.e. that the spatial autocorrelation pattern is constant in each direction.

For spatially correlated variables, the empirical variogram shows increasing semi-variance with the distance between points. After a certain distance (i.e. the range), points are spatially independent, and the semi-variance converges to a value (i.e. the sill) beyond which additional distance does not affect the dissimilarity. The value of semi-variance at the intercept (i.e. the nugget) represents the amount of random variation that is not spatially correlated at very short distances [3].

1. Fitting a theoretical variogram function

A theoretical variogram function is fitted to the empirical variogram to ease the estimation of the weights. There are different types of theoretical variogram functions: gaussian, spherical, exponential, linear, Matérn, and Matérn with M. Stein's parameterization, among others. In this study, the choice of a variogram function was based on the best fit, i.e. fitting all functions and using the one that minimises the sum of squared error (SSE), as done in [4]. All parameters of the theoretical variogram function, i.e. the nugget, the sill, the range and the kappa (for Matérn models), were tuned using the Levenberg-Marquardt algorithm [2,5].

1. Minimising the prediction variance

Interpolation weights are computed by minimising the variance of the estimation error or prediction variance, which is given by $var\left[ \hat{z}_{0}-z_{0} \right] ADDIN ZOTERO\_ITEM CSL\_CITATION \{"citationID":"7Q6pRcpo","properties":\{"formattedCitation":"[1]","plainCitation":"[1]","noteIndex":0\},"citationItems":[\{"id":992,"uris":["http://zotero.org/users/10170906/items/FIG3SIQY"],"itemData":\{"id":992,"type":"book","edition":"v0.7","language":"en","publisher":"Zenodo","source":"Zotero","title":"Computational modelling of terrains","URL":"https://doi.org/10.5281/zenodo.3992107","author":[\{"family":"Ledoux","given":"Hugo"\},\{"family":"Ohori","given":"Ken Arroyo"\},\{"family":"Peters","given":"Ravi"\}],"issued":\{"date-parts":[["2020"]]\}\}\}],"schema":"https://github.com/citation-style-language/schema/raw/master/csl-citation.json"\}$[1]$:$

|  | $var\left[ \hat{z}_{0}-z_{0} \right]= -\sum_{j=1}^{n} \sum_{i=1}^{n} {w_{i}w_{j}\gamma(s}_{i}- s_{j})+2 \sum_{i=1}^{n} {w_{i}\gamma(s}_{i}- s_{0})- {\gamma(s}_{0}- s_{0})$ | (2) |
| --- | --- | --- |

where $\hat{z}_{0}$ is the estimation of the expected value of the random variable $z$ at location $s_{0}$, $z_{0}$ is the expected value of the random variable $z$ at location $s_{0}$, $n$ is the number of sample points, and $w_{i},w_{j}$ are the weights of sample points at locations $s_{i}$and$s_{j}.$

The prediction variance depends on (1) the configuration of the $n$ sample points around each unsampled location (first term in (2)) and (2) the semi-variance $\gamma(h)$ between the sample points and the target location (second term in (2)) [1]. The prediction variance at each location provides a way to assess the uncertainty of the predictions; when interpolated values are based on very close neighbours, the variance of the estimation error is small.

## Bayesian geostatistical models

INLA assumes that behind each geostatistical process $z(s)$ there is an underlying stationary Gaussian random field, or Gaussian Process (GP), which represents the correlated spatial variation [6]. By approximating the GP with a Gaussian Markov random field (GMRF), INLA offers the advantage of working with a sparse precision matrix (due to Markov independence properties), which allows for efficient computations. A GP with Matérn covariance function is a solution to the following linear SPDE [7]:

|  | $\left( \kappa^{2}-\Delta\right)^{\frac{\alpha}{2}} u\left( s_{i} \right)=W\left( s_{i} \right), s_{i}\in\mathbb{R}^{d}, \alpha=\nu+d/2$ | (3) |
| --- | --- | --- |

where $\Delta$ is the Laplacian operator, $u\left( s_{i} \right)$ is the GP for locations $s_{i}\left( i=1,\ldots,n \right)$, and $W(s_{i})$ represents the Gaussian white noise.

This SPDE is solved using the finite element method, which tessellates the study area into a set of non-intersecting triangles, i.e. a mesh. The GP is approximated by a set of basis functions that are fitted to the mesh vertices [7]:

|  | $u\left( s_{i} \right)= \sum_{k=1}^{m} \psi_{k}\left( s_{i} \right)u_{k}$ | (4) |
| --- | --- | --- |

where $\psi_{k}\left( s_{i} \right)$ is the value of the basis function centred at mesh vertex $k(k=1,\ldots,m)$, and evaluated at location $s_{i}$ and $u_{k}$ is the value of the GP at vertex $k.$

In this paper, we used $1$ for the smoothness parameter $\nu$ ($\alpha=2)$. The range $r$ and the marginal standard deviation $\sigma_{u}$ of the GP were assigned Penalized Complexity priors such that:

|  | $P\left( r<6.46 \right)=0.99$  $P\left( \sigma_{u}>10 \right)=0.01$ | (5) |
| --- | --- | --- |

Note that 6.46 is the maximum distance (in degrees) measured across the study area. The remaining model parameters were assigned the default priors of INLA: the intercept has a Gaussian prior distribution ($N(0,0))$, the regression coefficients are assigned a Gaussian prior with zero mean and precision 0.001, and the precision $\sigma_{e}^{-2}$ of the uncorrelated residual term (for Gaussian likelihood) has a Gamma prior with parameters 1 and 0.0005.

# References

1. Ledoux H, Ohori KA, Peters R. Computational modelling of terrains. Zenodo; 2020.

2. Pebesma EJ. Multivariable geostatistics in S: the gstat package. Computers & Geosciences. 2004 Aug 1;30(7):683–91.

3. Gething P, Tatem A, Bird T, Burgert-Brucker CR. Creating Spatial Interpolation Surfaces with DHS Data. Rockville, Maryland, USA: ICF International; 2015. (DHS Spatial Analysis Reports No. 11).

4. Guisande C, Rueda-Quecho AJ, Rangel-Silva FA, Heine J, García-Roselló E, González-Dacosta J, et al. SINENVAP: An algorithm that employs kriging to identify optimal spatial interpolation models in polygons. Ecological Informatics. 2019 Sep;53:100975.

5. Moré JJ. The Levenberg-Marquardt algorithm: Implementation and theory. In: Watson GA, editor. Numerical Analysis. Berlin, Heidelberg: Springer; 1978. p. 105–16. (Lecture Notes in Mathematics).

6. Rue H, Martino S, Chopin N. Approximate Bayesian inference for latent Gaussian models by using integrated nested Laplace approximations. Journal of the Royal Statistical Society: Series B (Statistical Methodology). 2009;71(2):319–92.

7. Lindgren F, Rue H, Lindström J. An Explicit Link between Gaussian Fields and Gaussian Markov Random Fields: The Stochastic Partial Differential Equation Approach. Journal of the Royal Statistical Society Series B: Statistical Methodology. 2011 Sep 1;73(4):423–98.
