# Supplementary material for: Spatial interpolation of health and demographic variables: Predicting malaria indicators with and without covariates
Source: PLoS One. 2025 May 29;20(5):e0322819. doi: 10.1371/journal.pone.0322819 (PMC12121779; doi:10.1371/journal.pone.0322819)
Supplement: S1 Table — (DOCX) [file pone.0322819.s003.docx]

| CV RMSE | Indicator | IDW | TPS | OK | UK | RF | BM |
| --- | --- | --- | --- | --- | --- | --- | --- |
|  | Fula ethnicity | 0.207 | **0.194** | **0.194** | 0.205 | 0.209 | 0.226 |
|  | Stunting | 0.092 | 0.095 | 0.093 | 0.096 | **0.087** | 0.103 |
|  | Anemia | 0.128 | 0.126 | 0.126 | **0.122** | **0.122** | 0.125 |
|  | Sanitation | 0.213 | 0.207 | 0.208 | 0.188 | **0.163** | 0.174 |
|  | Wealth index | 0.649 | 0.623 | 0.564 | 0.518 | **0.330** | 0.488 |
|  | Literacy | 0.179 | 0.185 | 0.174 | 0.143 | 0.153 | **0.141** |
|  | ITN ownership | 0.109 | **0.100** | 0.116 | 0.103 | 0.106 | 0.101 |
|  | ITN ownership for 2 | 0.156 | 0.151 | 0.155 | 0.152 | 0.160 | **0.149** |
|  | ITN access | 0.128 | **0.125** | 0.136 | 0.133 | 0.134 | 0.138 |
|  | IRS | 0.128 | 0.124 | 0.119 | **0.116** | 0.143 | **0.116** |
| CV MAE | **Indicator** | **IDW** | **TPS** | **OK** | **UK** | **RF** | **BM** |
|  | Fula ethnicity | 0.162 | **0.150** | 0.152 | 0.162 | 0.161 | 0.174 |
|  | Stunting | 0.071 | 0.071 | 0.071 | 0.073 | **0.068** | 0.076 |
|  | Anemia | 0.101 | 0.101 | 0.100 | **0.096** | 0.098 | 0.099 |
|  | Sanitation | 0.170 | 0.168 | 0.171 | 0.159 | **0.129** | 0.139 |
|  | Wealth index | 0.500 | 0.479 | 0.434 | 0.413 | **0.258** | 0.392 |
|  | Literacy | 0.141 | 0.147 | 0.137 | 0.111 | 0.120 | **0.105** |
|  | ITN ownership | 0.074 | 0.068 | 0.079 | 0.069 | 0.070 | **0.067** |
|  | ITN ownership for 2 | 0.122 | **0.118** | 0.123 | 0.120 | 0.128 | 0.120 |
|  | ITN access | **0.098** | 0.099 | 0.105 | 0.102 | 0.105 | 0.107 |
|  | IRS | **0.044** | 0.060 | 0.052 | 0.059 | 0.064 | 0.060 |
| Training MAE | **Indicator** | **IDW** | **TPS** | **OK** | **UK** | **RF** | **BM** |
|  | Fula ethnicity | 0 | 0.113 | 1.03E-14 | 2.03E-15 | 0.083 | 0.035 |
|  | Stunting | 0 | 0.070 | 1.07E-14 | 6.90E-18 | 0.047 | 0.051 |
|  | Anemia | 0 | 0.091 | 8.08E-15 | 1.72E-16 | 0.082 | 0.074 |
|  | Sanitation | 0 | 0.133 | 1.53E-14 | 2.61E-16 | 0.093 | 0.069 |
|  | Wealth index | 0 | 0.370 | 2.03E-15 | 4.48E-16 | 0.182 | 0.254 |
|  | Literacy | 0 | 0.125 | 3.71E-16 | 1.81E-17 | 0.078 | 0.053 |
|  | ITN ownership | 0 | 0.041 | 1.81E-17 | 1.03E-16 | 0.040 | 0.046 |
|  | ITN ownership for 2 | 0 | 0.111 | 1.08E-16 | 5.41E-17 | 0.092 | 0.076 |
|  | ITN access | 0 | 0.051 | 1.02E-17 | 7.11E-18 | 0.058 | 0.031 |
|  | IRS | 0 | 0.043 | 7.03E-15 | 5.04E-16 | 0.036 | 0.027 |

*Note*. CV RMSE and CV MAE values are evaluated on the test set (20% of the data), while the training MAE is calculated based on the residuals from the model fit. Lower CV RMSE and CV MAE values indicate better model performance in cross-validation (CV). The best RMSE and MAE values are bold and underlined. Note that the training MAE should be compared with caution between methods as it is influenced by method assumptions (e.g. IDW has 0 as MAE because the interpolant is exact, i.e. passes exactly through the sample points). The vertical dotted line separates models that do not use covariates (left) from covariate-based models (right). The horizontal dotted lines separate socioeconomic indicators (up) and malaria prevention indicators (bottom). Abbreviations: CV (cross-validation), RMSE (root mean square error), MAE (mean absolute error), IDW (inverse distance weighting), TPS (thin plate spline), OK (ordinary kriging), UK (universal kriging), RF (random forest), BM (Bayesian model), ITN (insecticide-treated net), IRS (indoor residual spraying).
