# Supplementary material for: Spatial interpolation of health and demographic variables: Predicting malaria indicators with and without covariates
Source: PLoS One. 2025 May 29;20(5):e0322819. doi: 10.1371/journal.pone.0322819 (PMC12121779; doi:10.1371/journal.pone.0322819)
Supplement: S2 Table — (DOCX) [file pone.0322819.s004.docx]

| **Covariate** | **Fula ethnicity** | **Stunting** | **Anemia** | **Sanitation** | **Wealth index** | **Literacy** | **ITN ownership** | **ITN ownership for 2** | **ITN access** | **IRS** |
| --- | --- | --- | --- | --- | --- | --- | --- | --- | --- | --- |
| Precipitation |  | ○ |  |  | ○ |  | ○ |  | ○ | ○ |
| Temperature at 2 m | ○ | ○ | ○ | ○ | ○ |  | ○ | ○ | ○ | ○ |
| Potential evapotranspiration |  | ○ | ○ |  | ○ |  | ○ | ○ | ○ |  |
| Near-surface relative humidity | ○ | ○ | ○ | ○ | ○ | ○ | ○ | ○ | ○ | ○ |
| CMI |  | ○ |  |  | ○ |  | ○ |  | ○ | ○ |
| Day LST |  | ⨂ |  |  | ○ |  | ○ |  | ○ |  |
| Night LST | ⨯ | ⨯ |  |  |  |  | ○ |  | ○ |  |
| Daily LST range |  | ○ |  |  | ○ | ○ | ○ |  | ○ |  |
| NDVI |  | ○ | ○ |  | ○ |  | ○ |  | ○ |  |
| NDWI |  |  | ○ |  | ○ |  | ○ |  | ○ |  |
| NDMI | ○ | ○ |  |  | ○ | ○ | ○ |  | ○ |  |
| Prop. of water | ⨯ | ⨂ |  |  |  |  |  |  | ○ |  |
| Dist. to water |  | ○ | ○ |  | ○ | ○ | ○ |  | ○ | ⨯ |
| Prop. of trees |  | ○ | ⨂ |  | ○ |  | ○ |  | ○ |  |
| Dist. to trees |  | ○ | ○ | ⨯ |  | ⨯ | ○ |  | ○ |  |
| Prop. of flooded vegetation | ⨯ | ⨯ |  | ⨯ |  | ⨯ |  | ⨯ | ○ |  |
| Dist. to flooded vegetation |  |  |  | ⨯ | ○ |  | ⨂ |  | ⨂ | ⨯ |
| Prop. of crops | ⨂ | ⨂ | ⨯ | ⨯ |  | ⨯ | ⨂ | ⨯ | ○ | ⨯ |
| Dist. to crops |  |  | ⨯ |  |  | ⨯ | ○ |  | ○ | ⨯ |
| Prop. of grass |  |  |  |  |  |  |  | ⨯ | ○ | ⨯ |
| Dist. to grass |  |  | ⨯ | ⨯ | ⨯ | ⨯ | ⨂ |  | ○ |  |
| Prop. of bare ground |  | ○ |  |  | ○ |  | ○ |  | ○ |  |
| Dist. to bare ground | ⨯ | ⨂ |  |  | ○ |  | ○ | ⨯ | ○ |  |
| Prop. of shrubland | ⨯ | ⨯ | ○ | ⨯ | ○ |  | ⨂ |  | ○ | ⨯ |
| Dist. to shrubland | ⨯ |  |  |  | ○ |  | ○ |  | ○ |  |
| Prop. of settlements |  | ○ | ○ |  | ○ |  | ○ | ○ | ○ |  |
| Dist. to settlements | ⨯ | ⨂ | ○ |  | ○ | ⨯ | ○ | ⨯ | ⨂ |  |
| Residential built-up surface |  | ○ | ○ | ○ | ○ | ○ | ○ | ○ | ○ | ⨯ |
| Building height |  | ○ | ○ |  | ○ |  | ○ | ○ | ○ |  |
| Average nighttime lights |  | ○ | ○ | ○ | ○ |  | ○ | ○ | ○ |  |
| Median nighttime lights |  | ○ | ○ | ○ | ○ |  | ○ | ○ | ○ |  |
| Dist. to major roads |  | ○ | ○ | ⨯ | ⨂ | ⨂ | ○ | ○ | ⨂ | ⨯ |
| Dist. to waterways |  |  | ⨯ |  | ○ | ⨯ | ⨯ |  | ○ |  |
| Dist. to education |  | ○ | ○ |  | ○ | ⨂ | ○ |  | ○ | ⨯ |
| Dist. to health facilities | ○ | ⨂ | ○ |  | ○ | ○ | ○ | ○ | ○ |  |
| Travel time to cities |  | ○ | ○ | ○ | ○ | ⨂ | ○ | ○ | ○ |  |
| Travel time to healthcare (walking-only) | ⨯ | ⨂ | ○ | ⨯ | ⨂ | ⨂ | ○ | ⨂ | ○ | ⨯ |
| Travel time to healthcare (motorized) |  | ○ | ○ |  | ⨂ | ○ | ○ | ○ | ○ | ⨯ |
| Population counts | ○ | ○ | ○ | ○ | ○ | ○ | ⨂ | ○ | ○ |  |
| Births | ○ | ○ | ○ | ○ | ○ |  | ○ | ○ | ○ |  |
| Pregnancies | ○ | ○ | ○ | ○ | ○ |  | ○ | ○ | ○ |  |
| Prop. of Wolof | Not tested | ○ |  | ○ | ○ |  | ○ |  | ○ | ⨂ |
| Prop. of Fula | Not tested | ⨂ |  | ○ | ⨂ |  | ○ |  | ○ |  |
| Prop. of Serer | Not tested | ○ | ⨯ | ⨯ | ○ | ⨯ | ⨂ | ⨯ | ○ | ○ |
| Prop. of Diola | Not tested |  | ⨂ | ⨯ | ⨯ | ⨂ | ○ |  | ○ | ⨯ |
| Prop. of Mandingue | Not tested | ○ |  | ⨯ |  |  | ⨂ |  | ⨂ |  |
| Prop. of Soninke | Not tested |  | ⨯ |  |  |  | ⨂ |  | ⨂ | ⨯ |
| Prop. of not Senegalese | Not tested |  |  |  |  |  | ⨂ | ⨯ | ⨂ | ⨯ |
| Prop. of other ethnic groups | Not tested |  |  | ⨯ |  |  | ⨂ | ⨯ | ⨂ |  |
| Elevation | ⨯ | ⨂ | ⨯ | ○ | ○ | ⨂ | ○ | ⨯ | ⨂ |  |
| Density of goat |  |  |  |  |  |  | ○ |  | ○ |  |
| Density of cattle |  |  |  |  |  | ⨯ | ⨂ |  | ○ |  |
| Density of pig | ○ |  |  |  | ○ |  | ○ | ⨯ | ○ |  |
| Density of poultry |  | ○ | ○ |  |  | ⨯ | ⨂ | ⨯ | ⨂ |  |
| Density of sheep | ○ |  | ⨯ |  |  | ⨯ | ○ |  | ○ |  |
| Latitude |  | ○ | ○ |  | ○ |  | ○ |  | ○ | ○ |
| Longitude | ○ | ○ | ○ | ○ | ○ | ○ | ○ | ○ | ○ | ○ |
| Total number of covariates for RF | 11 | 39 | 28 | 13 | 40 | 15 | 53 | 17 | 57 | 8 |
| Total number of covariates for UK & BM | 10 | 12 | 10 | 12 | 6 | 17 | 13 | 13 | 9 | 15 |
|  | ○ selected for RF; ⨯ selected for UK & BM; ⨂ selected for all (RF, UK & BM) | | | | | | | | | |

*Note.* RF models used a recursive feature elimination to select covariates. For UK and BM, the best set of covariates was selected by fitting linear regression models and implementing stepwise feature selection. From this selected set, only covariates with VIF (Variance Inflation Factor) below 5 were retained. Latitude and longitude are only inputs for covariate selection for RF models as they do not explicitly account for spatial autocorrelation, compared to UK and BM. Ethnicity-related variables (proportion of Wolof, Fula, Serer, Diola, Mandingue, Soninke and others) were not used to model the proportion of Fula people (to avoid circularity). Abbreviations: UK (universal kriging), RF (random forest), BM (Bayesian model), ITN (insecticide-treated net), IRS (indoor residual spraying), prop. (proportion), dist. (distance), DHS (Demographic and Health Surveys).
