# Supplementary material for: Spatial interpolation of health and demographic variables: Predicting malaria indicators with and without covariates
Source: PLoS One. 2025 May 29;20(5):e0322819. doi: 10.1371/journal.pone.0322819 (PMC12121779; doi:10.1371/journal.pone.0322819)
Supplement: S3 Table — (DOCX) [file pone.0322819.s005.docx]

| IDW | Description of parameter | Fula ethnicity | Stunting | | Anemia | | Sanitation | | Wealth index | | Literacy | | ITN ownership | | | ITN ownership for 2 | | ITN access | | IRS |
| --- | --- | --- | --- | --- | --- | --- | --- | --- | --- | --- | --- | --- | --- | --- | --- | --- | --- | --- | --- | --- |
| $\boldsymbol{p}$ | power to distance | 0.5 | 1 | | 1 | | 1.5 | | 3 | | 2 | | 2 | | | 0.5 | | 1.5 | | 3 |
| $\boldsymbol{k}$ | number of neighbours | 6 | 36 | | 31 | | 31 | | 16 | | 11 | | 46 | | | 31 | | 21 | | 1 |
| TPS | Description of parameter | Fula ethnicity | Stunting | | Anemia | | Sanitation | | Wealth index | | Literacy | | ITN ownership | | | ITN ownership for 2 | | ITN access | | IRS |
| $\boldsymbol{\lambda}$ | penalty parameter | 5e-04 | 0.005 | | 0.005 | | 5e-04 | | 5e-04 | | 0.003 | | 5e-05 | | | 0.003 | | 3e-05 | | 3e-04 |
| OK | Description of parameter | Fula ethnicity | Stunting | | Anemia | | Sanitation | | Wealth index | | Literacy | | ITN ownership | | | ITN ownership for 2 | | ITN access | | IRS |
| cutoff | maximum distance in variogram (km) | 250 | 250 | | 150 | | 300 | | 300 | | 300 | | 400 | | | 400 | | 300 | | 150 |
| width | variogram bin width (km) | 26.67 | 13.33 | | 16.67 | | 30 | | 16.67 | | 16.67 | | 10 | | | 26.67 | | 6.67 | | 10 |
| function | variogram theoretical function | Stein | Stein | | Matérn | | Matérn | | Matérn | | Stein | | Stein | | | Matérn | | Spherical | | Matérn |
| $\boldsymbol{\kappa}$ | smoothness parameter (for Matérn and Stein models) | 0.5 | 0.5 | | 1 | | 0.3 | | 0.4 | | 0.3 | | 0.4 | | | 5 | | / | | 0.5 |
| UK | Description of parameter | Fula ethnicity | Stunting | | Anemia | | Sanitation | | Wealth index | | Literacy | | ITN ownership | | | ITN ownership for 2 | | ITN access | | IRS |
| cutoff | maximum distance in variogram (km) | 400 | 100 | | 350 | | 550 | | 400 | | 100 | | 200 | | | 300 | | 100 | | 300 |
| width | variogram bin width (km) | 33.33 | 16.67 | | 23.33 | | 16.67 | | 26.67 | | 40 | | 20 | | | 26.67 | | 6.67 | | 20 |
| function | variogram theoretical function | Matérn | Spherical | | Stein | | Stein | | Stein | | Spherical | | Stein | | | Linear | | Linear | | Stein |
| $\boldsymbol{\kappa}$ | smoothness parameter (for Matérn and Matérn with M. Stein's parameterization functions) | 1.4 | / | | 5 | | 5 | | 5 | | / | | 0.3 | | | / | | / | | 0.6 |
| RF | Description of parameter | Fula ethnicity | Stunting | | Anemia | | Sanitation | | Wealth index | | Literacy | | ITN ownership | | | ITN ownership for 2 | | ITN access | | IRS |
| frac | fraction of observation used per decision tree | 0.88 | 0.73 | | 0.20 | | 0.65 | | 0.72 | | 0.87 | | 0.80 | | | 0.37 | | 0.76 | | 0.82 |
| ntree | number of trees | 1588 | 1123 | | 1116 | | 901 | | 518 | | 1428 | | 639 | | | 1354 | | 952 | | 1131 |
| mtry | number of covariates at node split | 6 | 1 | | 25 | | 8 | | 4 | | 2 | | 7 | | | 1 | | 6 | | 1 |
| nodesize | minimum number of observations per terminal node | 5 | 9 | | 8 | | 10 | | 7 | | 1 | | 3 | | | 4 | | 1 | | 2 |
| BM | Description of parameter | Fula ethnicity | | Stunting | | Anemia | | Sanitation | | Wealth index | | Literacy | | ITN ownership | ITN ownership for 2 | | ITN access | | IRS | |
| likelihood | Likelihood function | Binomial | | Binomial | | Binomial | | Binomial | | Gaussian | | Binomial | | Binomial | Binomial | | Binomial | | Zero-inflated binomial | |
| $\boldsymbol{\nu}$ | Smoothness parameter in Matérn covariance function | 1 (i.e., $\alpha=2$) | | | | | | | | | | | | | | | | | | |
| $\boldsymbol{r}$ | Range | \|  \| \| --- \|   Penalized Complexity prior: $P\left( r<6.46 \right)= 0.99$, where 6.46 is the maximum distance (in degrees) measured across the study area | | | | | | | | | | | | | | | | | | |
| $\boldsymbol{\sigma}_{\boldsymbol{u}}$ | Marginal standard deviation of the Gaussian Process | Penalized Complexity prior: $P(\sigma_{u}>10) = 0.01$ | | | | | | | | | | | | | | | | | | |
| $\boldsymbol{\beta}_{\boldsymbol{0}}$ | Intercept | Gaussian prior: $N(0,0)$ | | | | | | | | | | | | | | | | | | |
| $\boldsymbol{\beta}$ | Regression coefficients | Gaussian prior: Mean = 0, Precision = 0.001 | | | | | | | | | | | | | | | | | | |
| $\boldsymbol{\sigma}_{\boldsymbol{e}}^{\boldsymbol{-2}}$ | Precision of uncorrelated residual term (for Gaussian likelihood) | Gamma prior: Parameters 1 and 0.0005 | | | | | | | | | | | | | | | | | | |

*Note.* The parameter values presented here correspond to the models used to assess performance on the test set. Further details on each parameter can be found in the method section. Abbreviations: IDW (inverse distance weighting), TPS (thin plate spline), OK (ordinary kriging), UK (universal kriging), RF (random forest), BM (Bayesian model), ITN (insecticide-treated net), IRS (indoor residual spraying), Stein (Matérn with M. Stein's parameterization).
