# Supplementary figures and images for: Spatial interpolation of health and demographic variables: Predicting malaria indicators with and without covariates
Source: PLoS One. 2025 May 29;20(5):e0322819. doi: 10.1371/journal.pone.0322819 (PMC12121779; doi:10.1371/journal.pone.0322819)

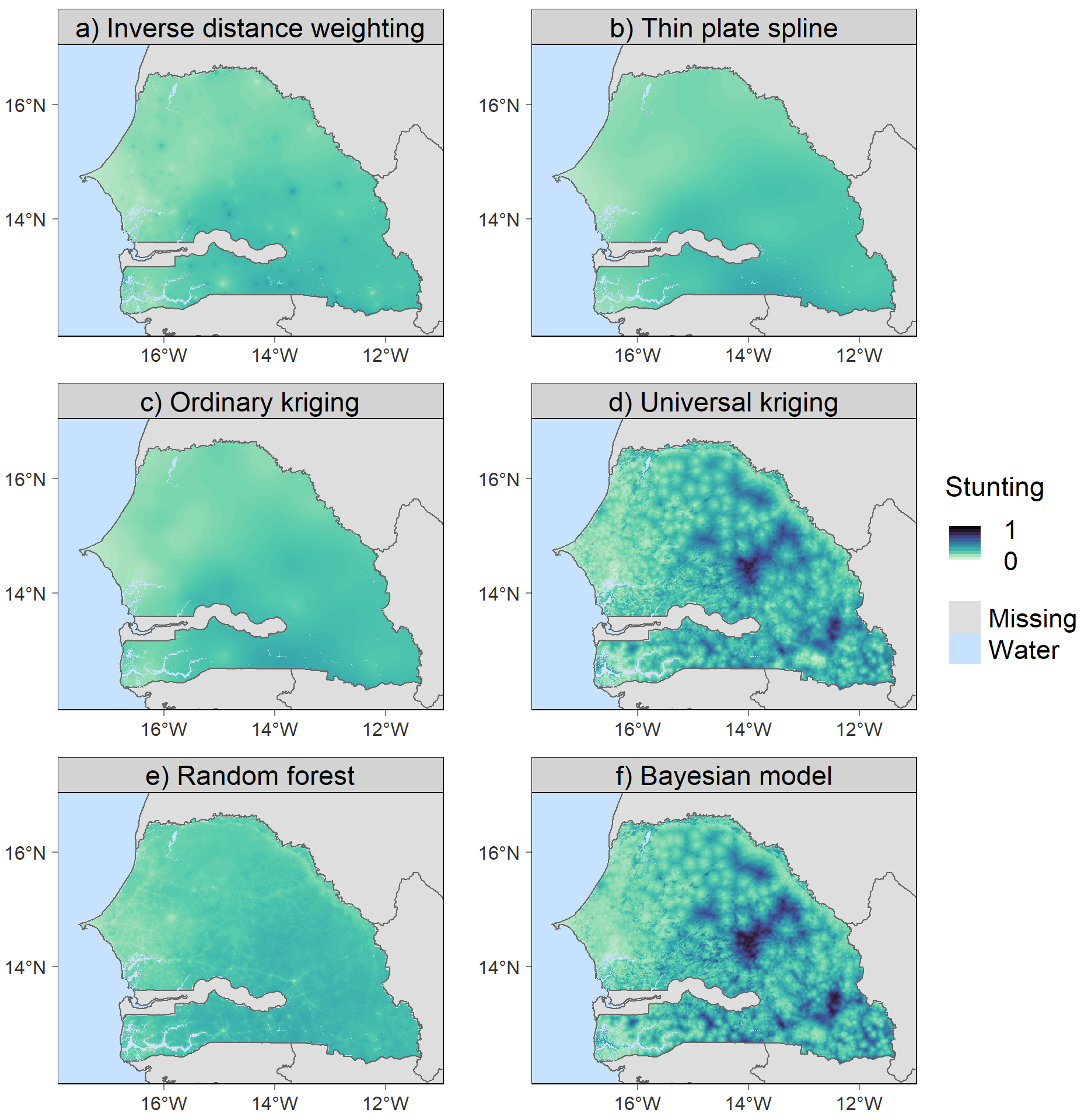

Supplement: S1 Fig — The maps show the spatial distribution of the proportion (ranging from 0 to 1) of children under 5 years old that are moderately or severely stunted in Senegal. National boundaries were downloaded from GADM. (TIF) [file pone.0322819.s006.tif]

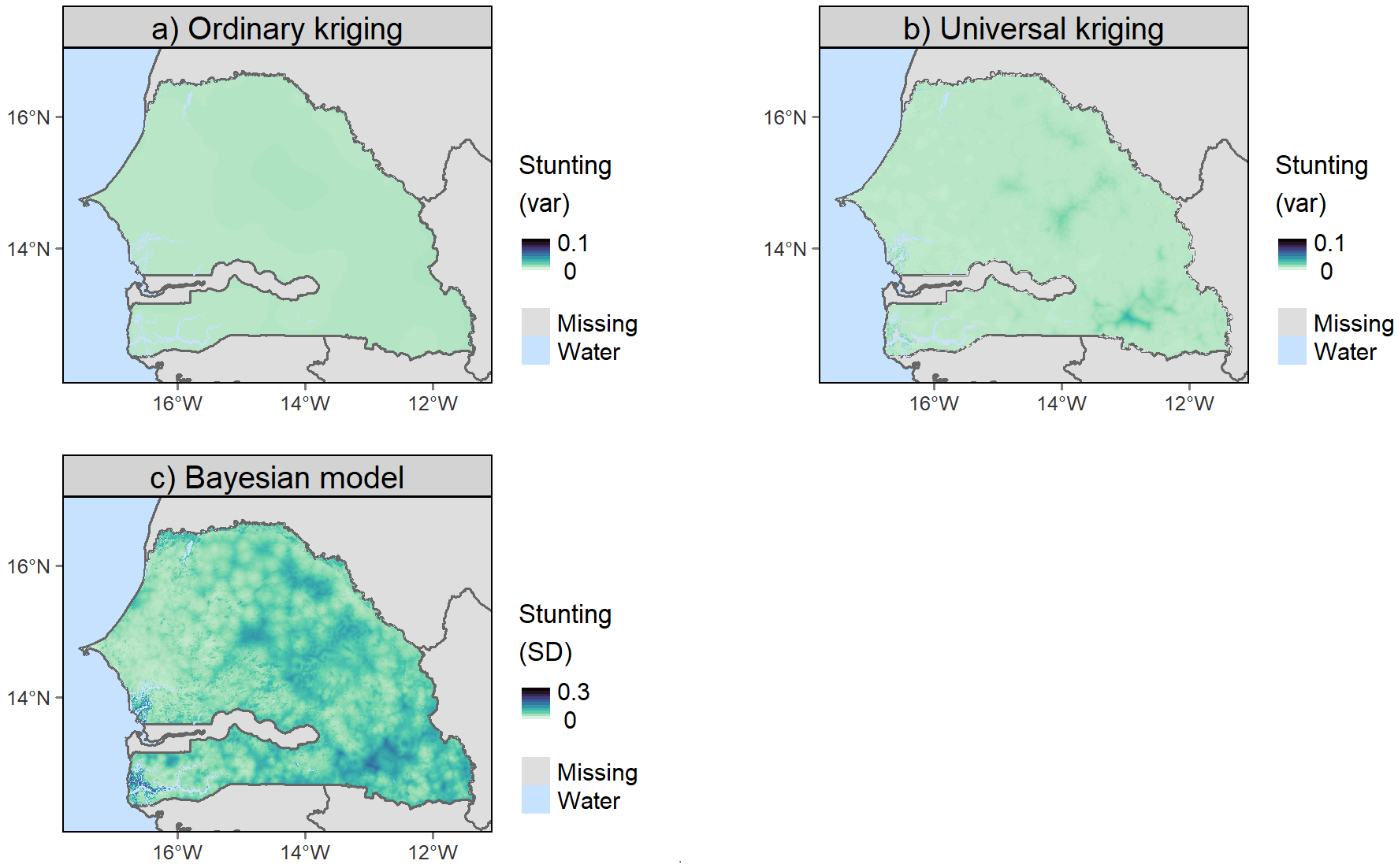

Supplement: S2 Fig — Uncertainty is measured as a prediction variance (var) for kriging methods (a, b) and as a standard deviation (SD) for Bayesian models (c). Higher values of SD or variance indicate areas with greater uncertainty in the predicted indicator, reflecting lower confidence in the accuracy of the predictions in these regions. National boundaries were downloaded from GADM. (TIF) [file pone.0322819.s007.tif]

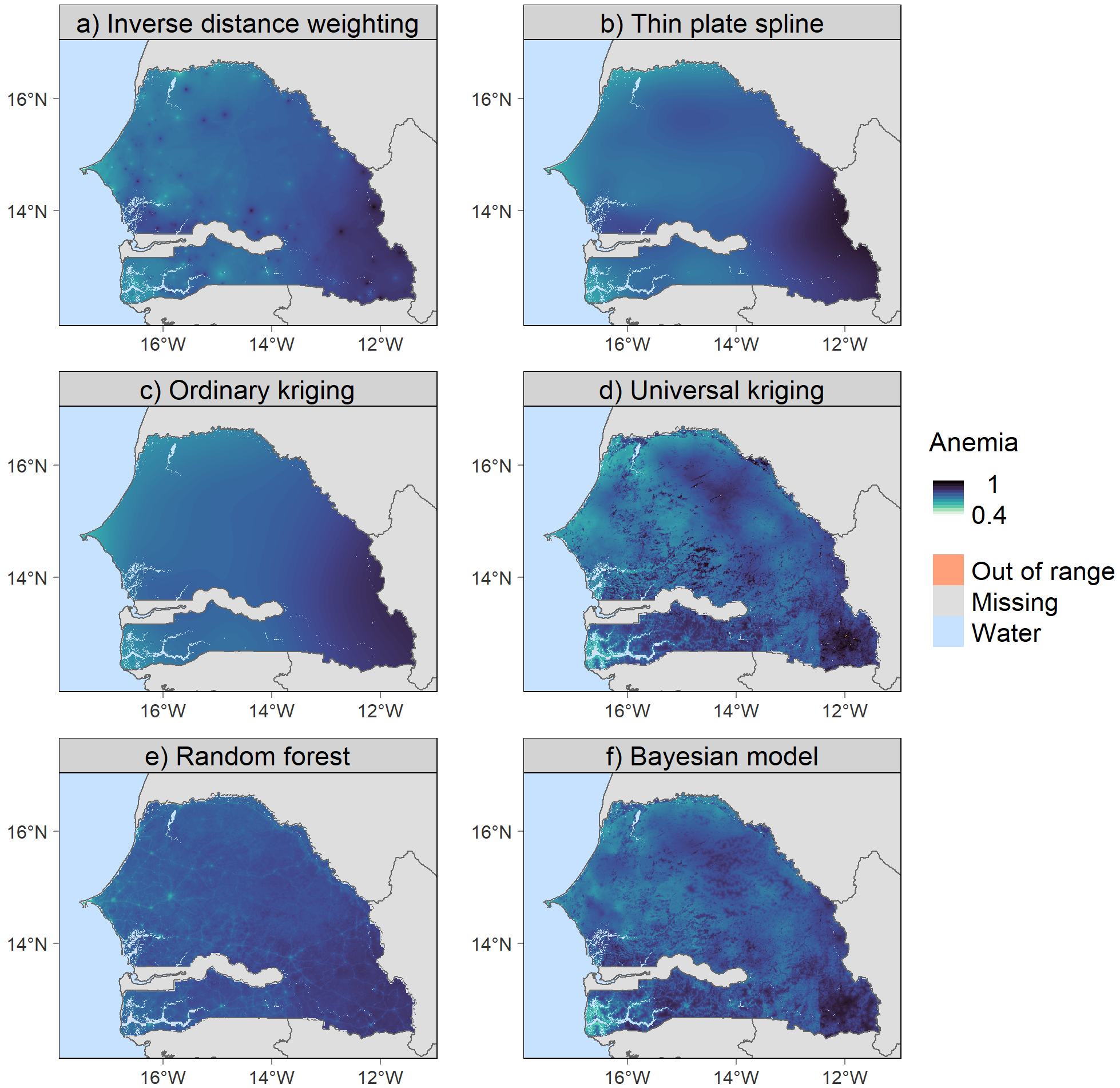

Supplement: S3 Fig — The maps show the spatial distribution of the proportion (ranging from 0 to 1) of children with mild, moderate or severe anemia in Senegal. Gridded surfaces are produced at a resolution of 1x1 km for all methods examined in the study. The ‘Out of range’ label indicates predicted values that are outside the possible range of values of the indicator (below 0 or above 1). Out-of-range predictions were made by universal kriging. National boundaries were downloaded from GADM. (TIF) [file pone.0322819.s008.tif]

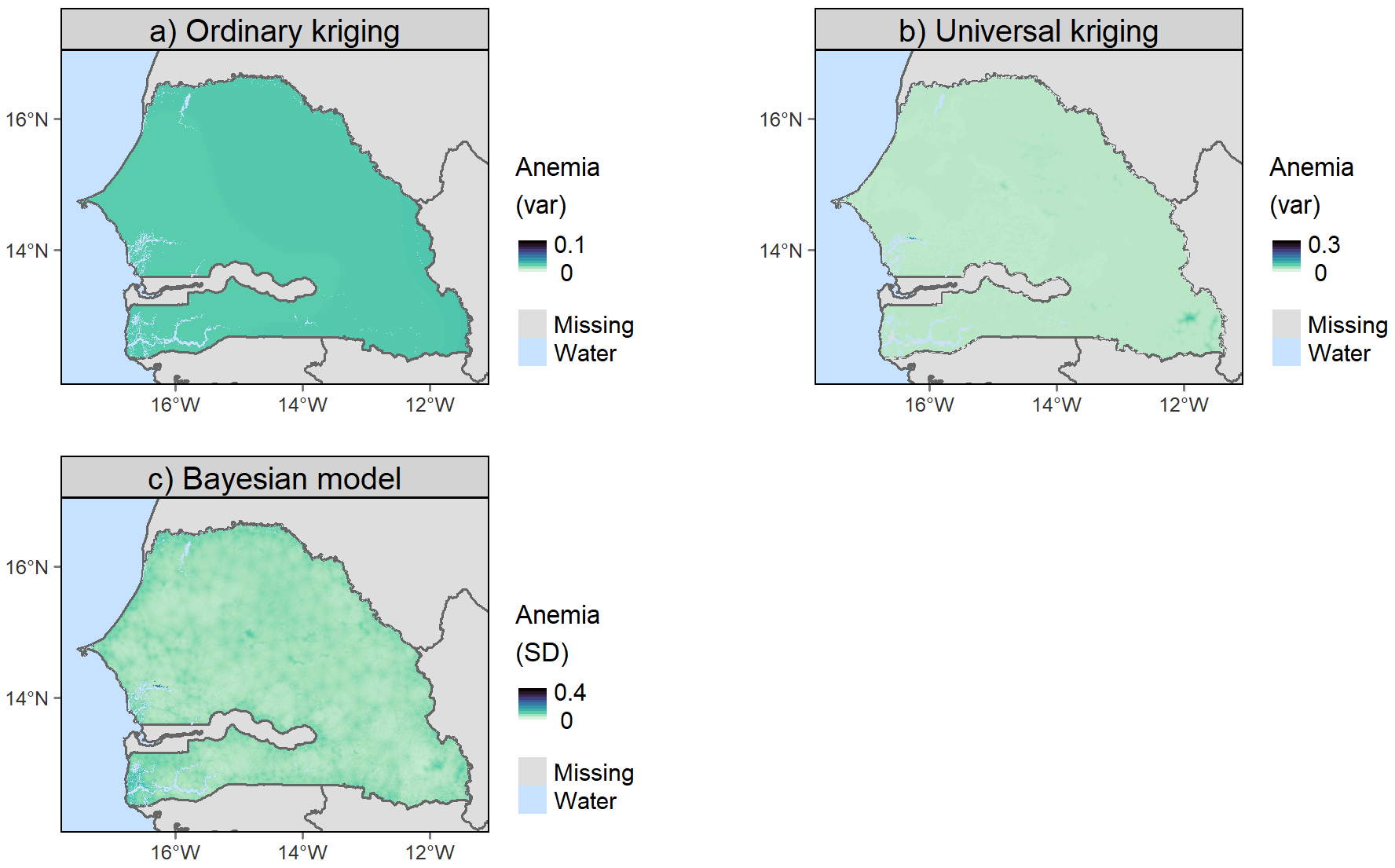

Supplement: S4 Fig — Uncertainty is measured as a prediction variance (var) for kriging methods (a, b) and as a standard deviation (SD) for Bayesian models (c). Higher values of SD or variance indicate areas with greater uncertainty in the predicted indicator, reflecting lower confidence in the accuracy of the predictions in these regions. National boundaries were downloaded from GADM. (TIF) [file pone.0322819.s009.tif]

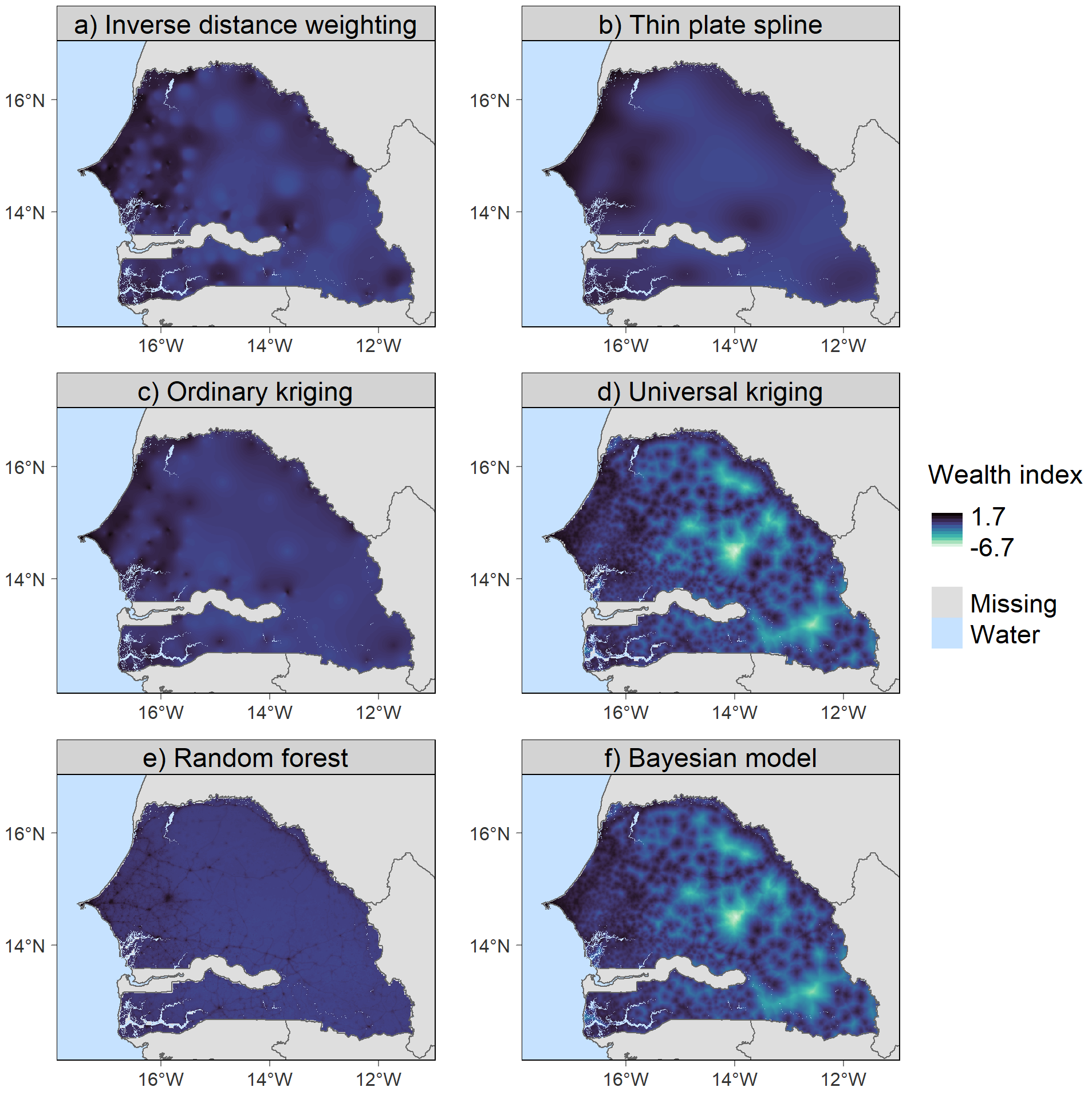

Supplement: S5 Fig — The maps show the spatial distribution of the household wealth index in Senegal. Gridded surfaces are produced at a resolution of 1x1 km for all methods examined in the study. National boundaries were downloaded from GADM. (TIF) [file pone.0322819.s010.tif]

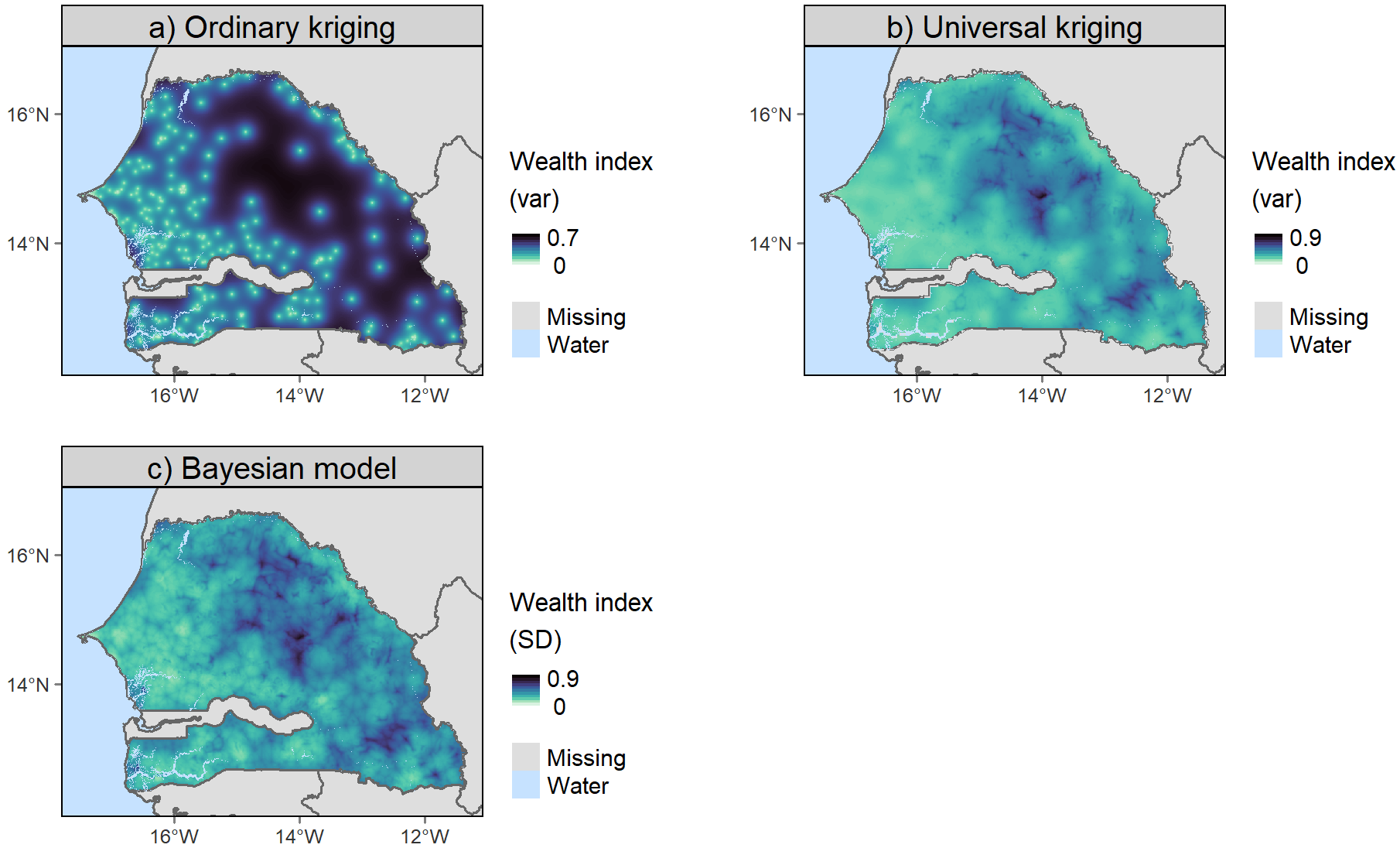

Supplement: S6 Fig — Uncertainty is measured as a prediction variance (var) for kriging methods (a, b) and as a standard deviation (SD) for Bayesian models (c). Higher values of SD or variance indicate areas with greater uncertainty in the predicted indicator, reflecting lower confidence in the accuracy of the predictions in these regions. National boundaries were downloaded from GADM. (TIF) [file pone.0322819.s011.tif]

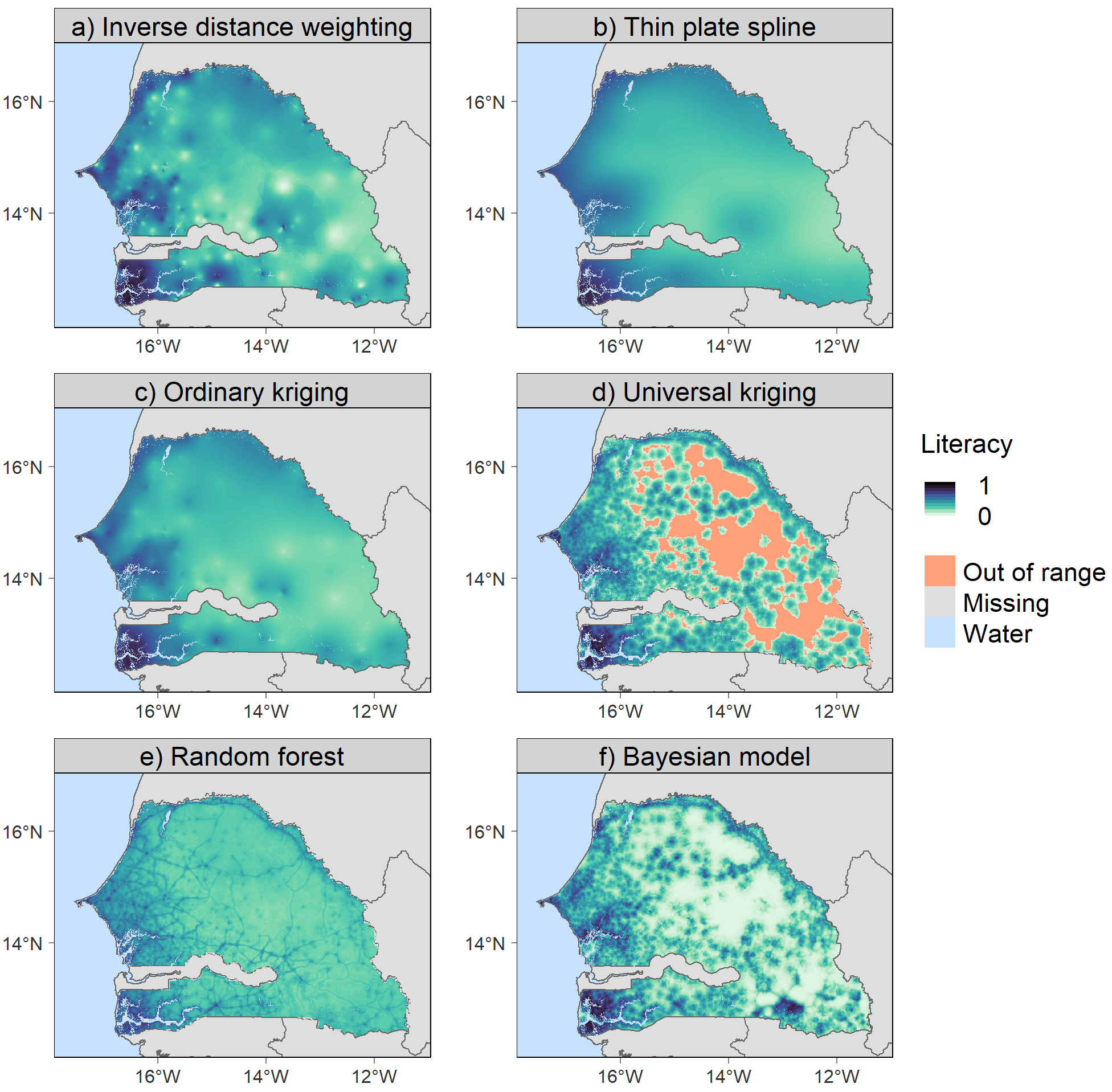

Supplement: S7 Fig — The maps show the spatial distribution of the proportion (ranging from 0 to 1) of women who are literate in Senegal. Gridded surfaces are produced at a resolution of 1x1 km for all methods examined in the study. The ‘Out of range’ label indicates predicted values that are outside the possible range of values of the indicator (below 0 or above 1). Out-of-range predictions were made by universal kriging. National boundaries were downloaded from GADM. (TIF) [file pone.0322819.s012.tif]

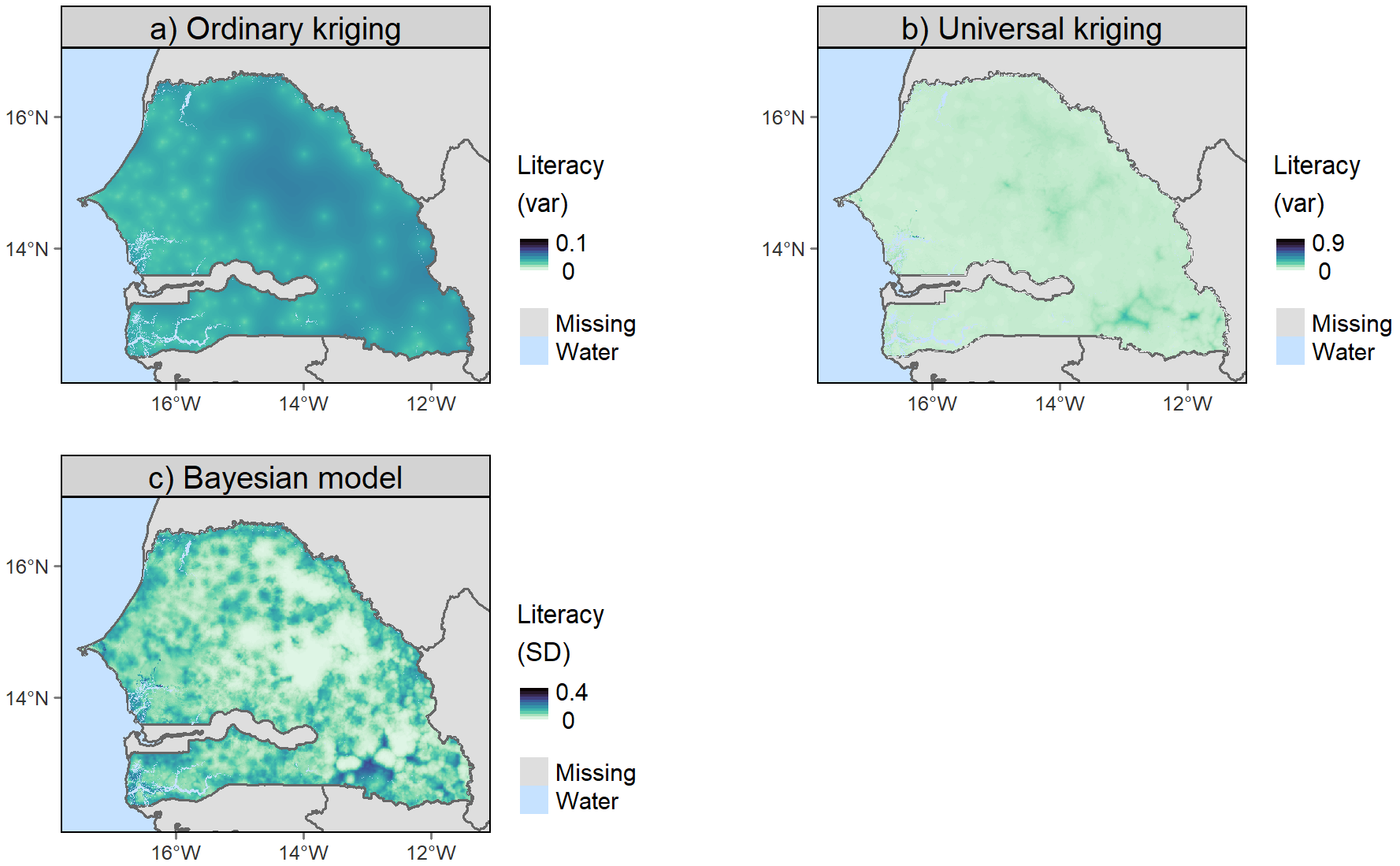

Supplement: S8 Fig — Uncertainty is measured as a prediction variance (var) for kriging methods (a, b) and as a standard deviation (SD) for Bayesian models (c). Higher values of SD or variance indicate areas with greater uncertainty in the predicted indicator, reflecting lower confidence in the accuracy of the predictions in these regions. National boundaries were downloaded from GADM. (TIF) [file pone.0322819.s013.tif]

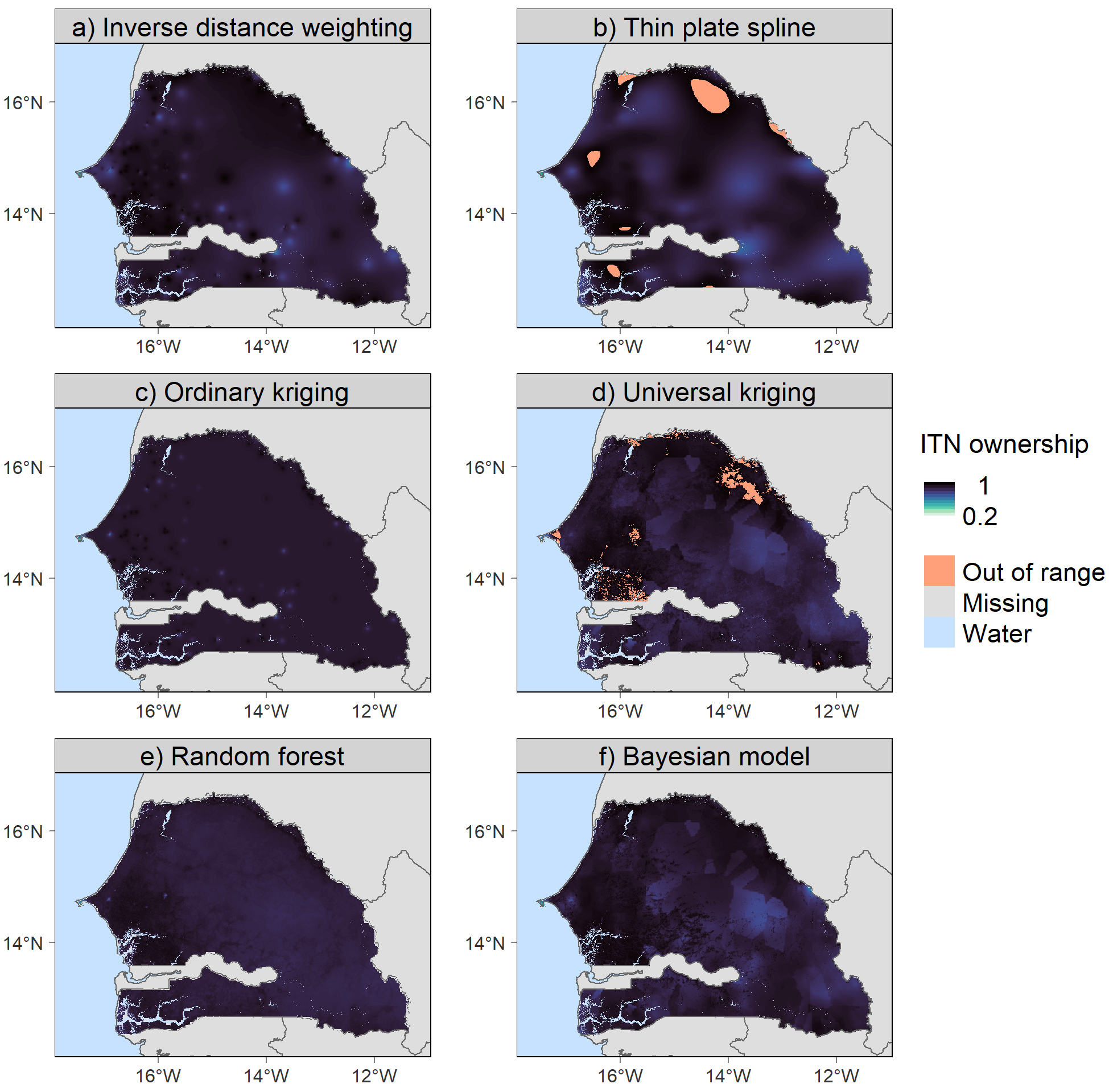

Supplement: S9 Fig — The maps show the spatial distribution of the proportion (ranging from 0 to 1) of households with at least one insecticide-treated net (ITN) in Senegal. Gridded surfaces are produced at a resolution of 1x1 km for all methods examined in the study. The ‘Out of range’ label indicates predicted values that are outside the possible range of values of the indicator (below 0 or above 1). Out-of-range predictions were made by thin plate spline and universal kriging. National boundaries were downloaded from GADM. (TIF) [file pone.0322819.s014.tif]

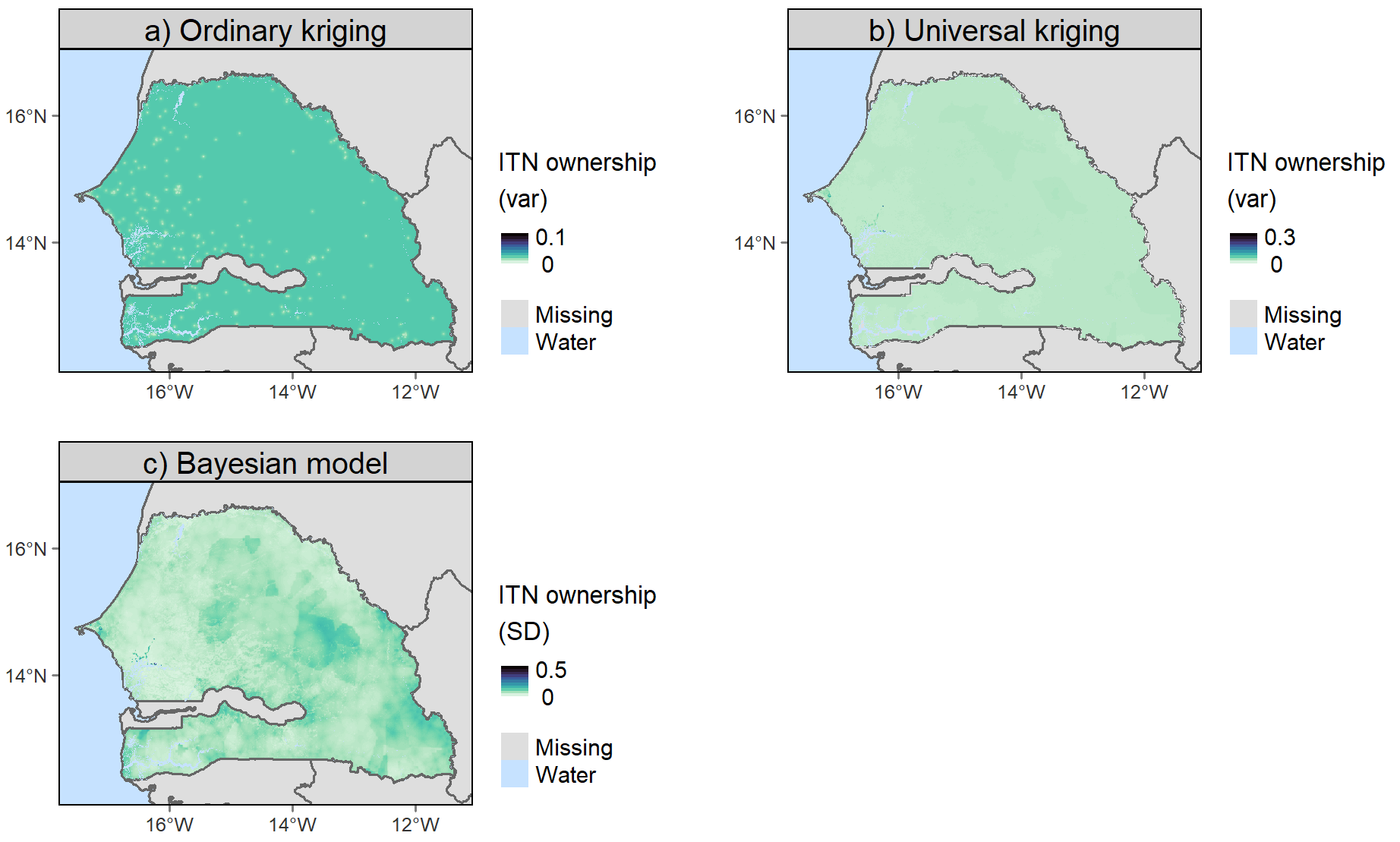

Supplement: S10 Fig — Uncertainty is measured as a prediction variance (var) for kriging methods (a, b) and as a standard deviation (SD) for Bayesian models (c). Higher values of SD or variance indicate areas with greater uncertainty in the predicted indicator, reflecting lower confidence in the accuracy of the predictions in these regions. ITN stands for insecticide-treated net. National boundaries were downloaded from GADM. (TIF) [file pone.0322819.s015.tif]

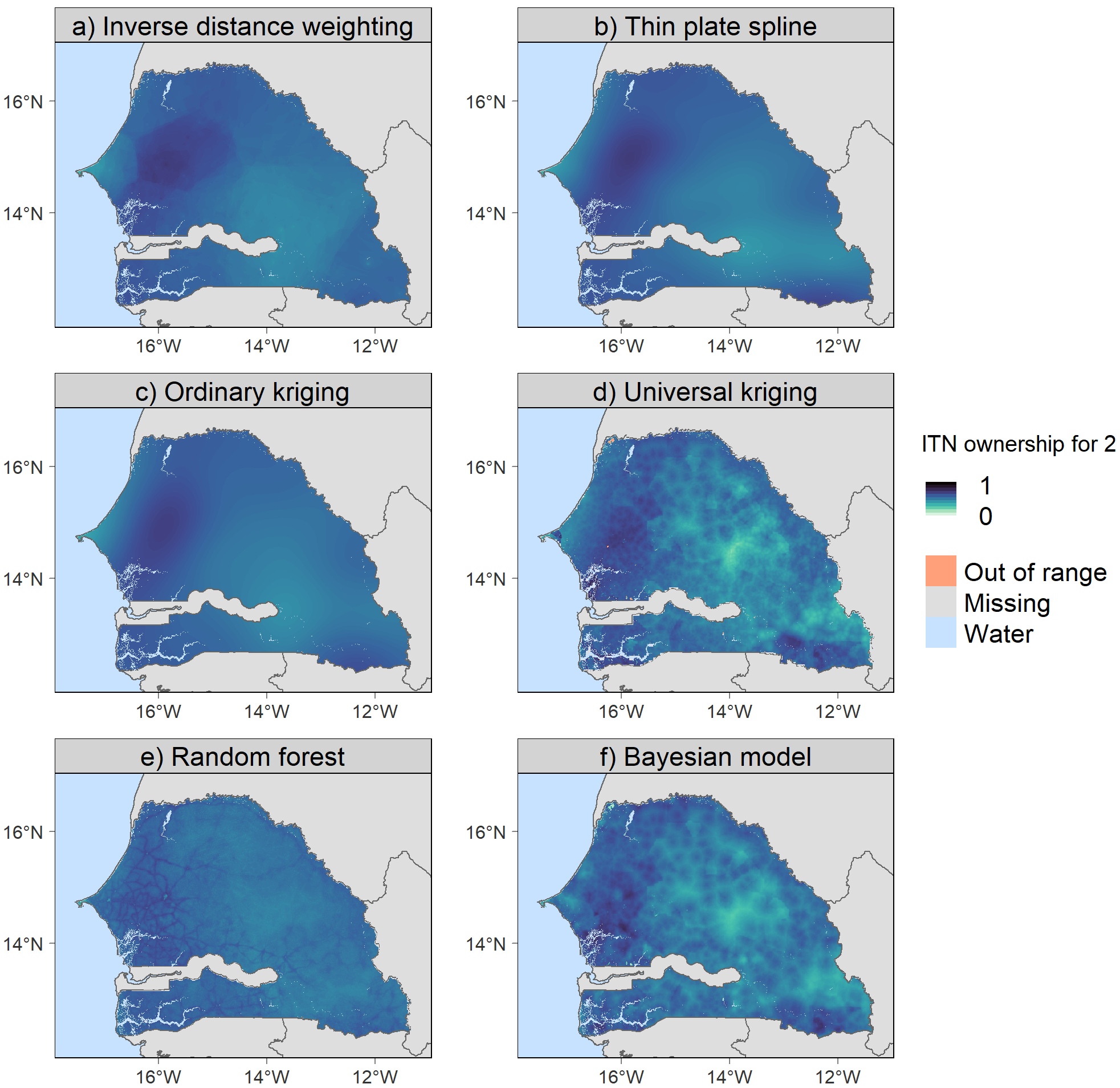

Supplement: S11 Fig — The maps show the spatial distribution of the proportion (ranging from 0 to 1) of households with at least one insecticide-treated net (ITN) for every two people who slept in the house the night before the survey. Gridded surfaces are produced at a resolution of 1x1 km for all methods examined in the study. The ‘Out of range’ label indicates predicted values that are outside the possible range of values of the indicator (below 0 or above 1). Out-of-range predictions were made by universal kriging. National boundaries were downloaded from GADM. (TIF) [file pone.0322819.s016.tif]

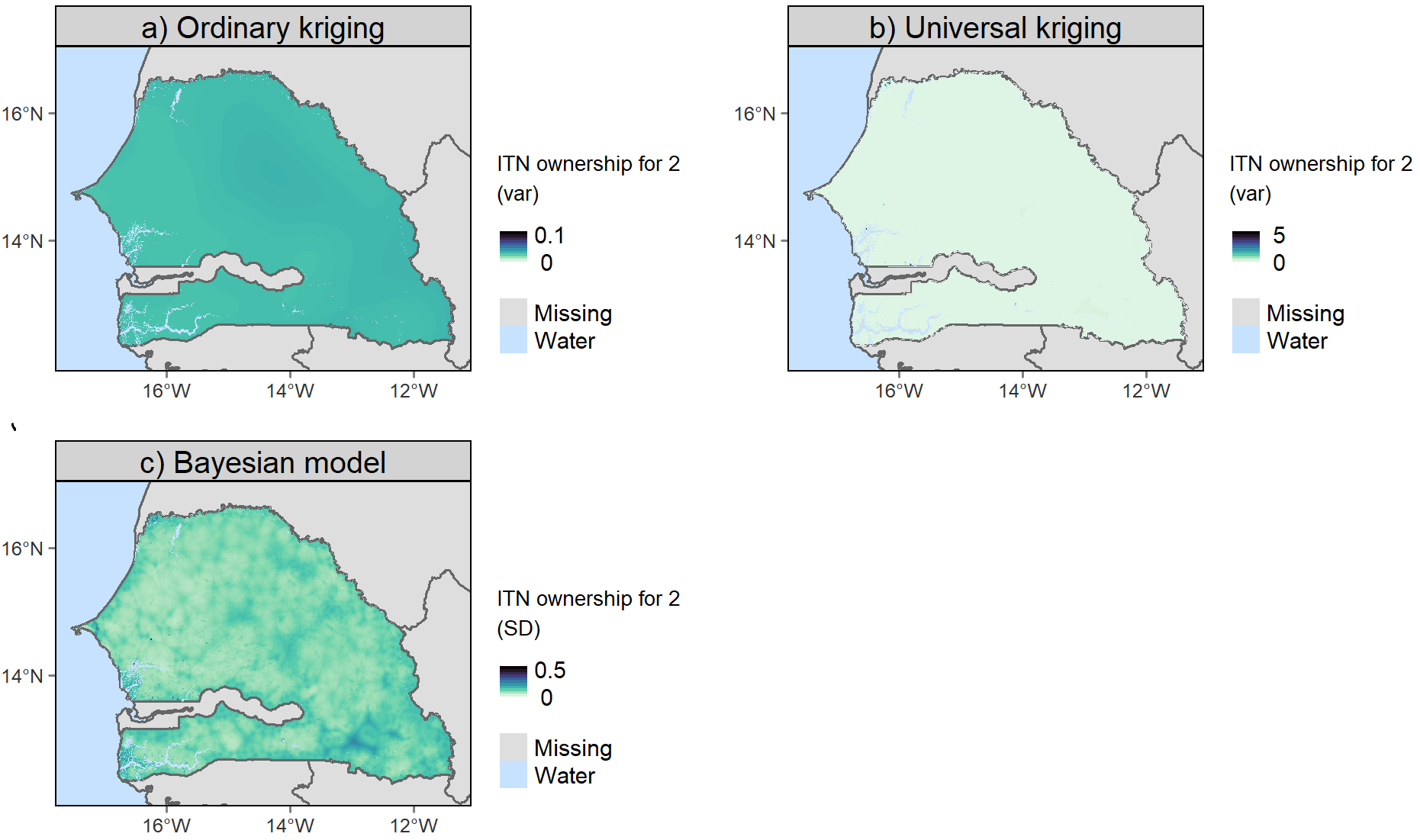

Supplement: S12 Fig — Uncertainty is measured as a prediction variance (var) for kriging methods (a, b) and as a standard deviation (SD) for Bayesian models (c). Higher values of SD or variance indicate areas with greater uncertainty in the predicted indicator, reflecting lower confidence in the accuracy of the predictions in these regions. ITN stands for insecticide-treated net. National boundaries were downloaded from GADM. (TIF) [file pone.0322819.s017.tif]

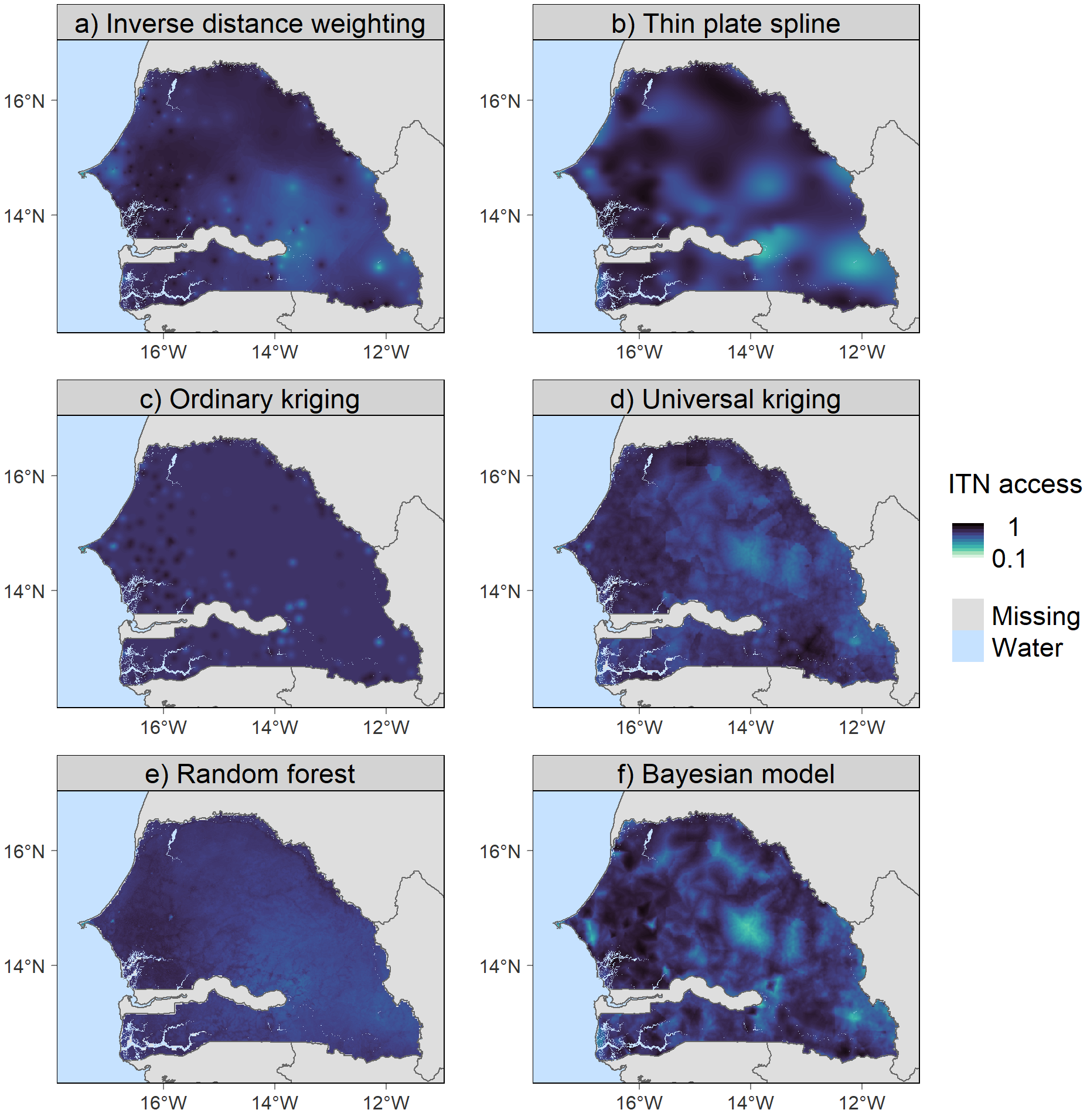

Supplement: S13 Fig — The maps show the spatial distribution of the proportion (ranging from 0 to 1) of population with access to an insecticide-treated net (ITN) in their household in Senegal. Gridded surfaces are produced at a resolution of 1x1 km for all methods examined in the study. National boundaries were downloaded from GADM. (TIF) [file pone.0322819.s018.tif]

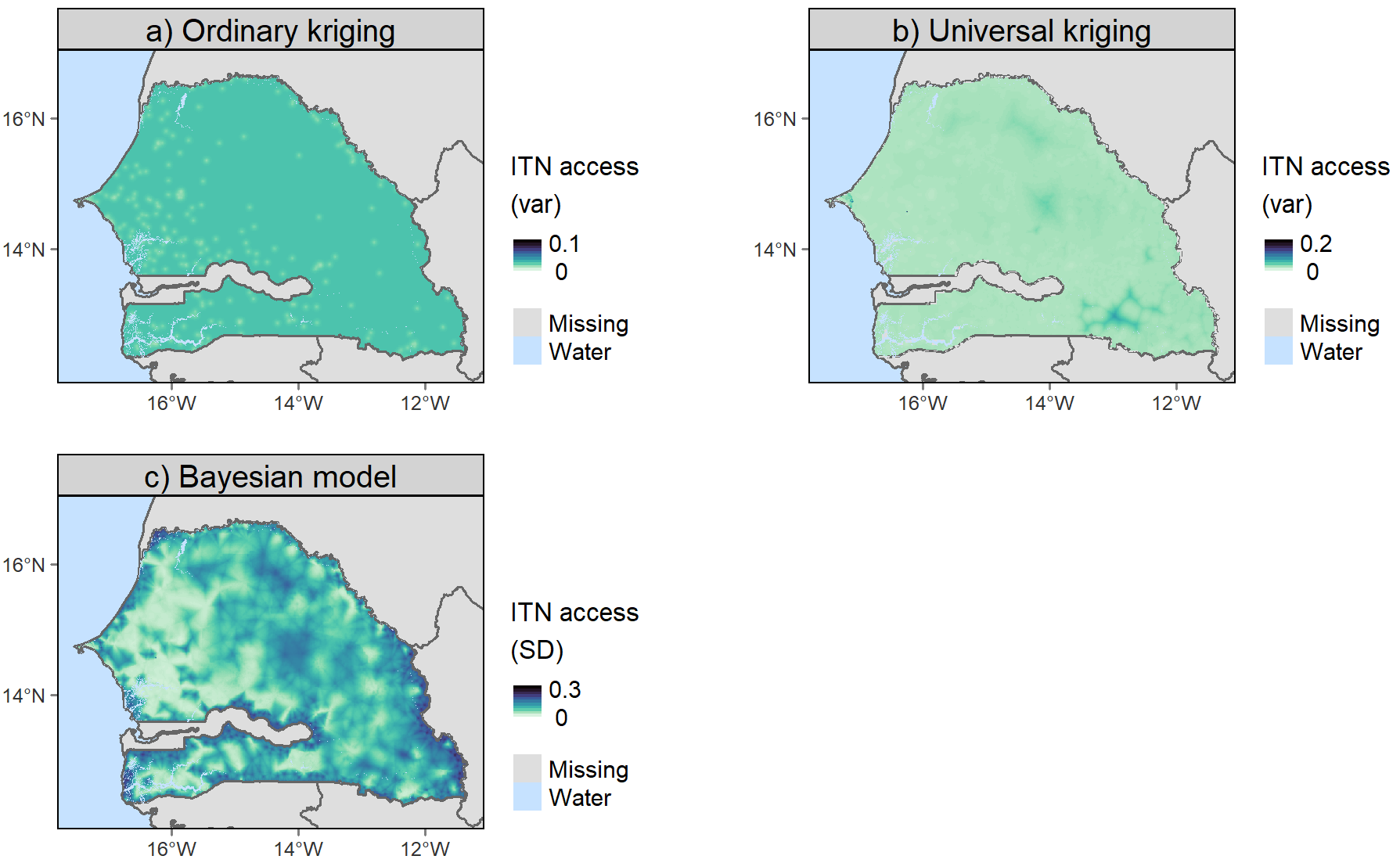

Supplement: S14 Fig — Uncertainty is measured as a prediction variance (var) for kriging methods (a, b) and as a standard deviation (SD) for Bayesian models (c). Higher values of SD or variance indicate areas with greater uncertainty in the predicted indicator, reflecting lower confidence in the accuracy of the predictions in these regions. ITN stands for insecticide-treated net. National boundaries were downloaded from GADM. (TIF) [file pone.0322819.s019.tif]

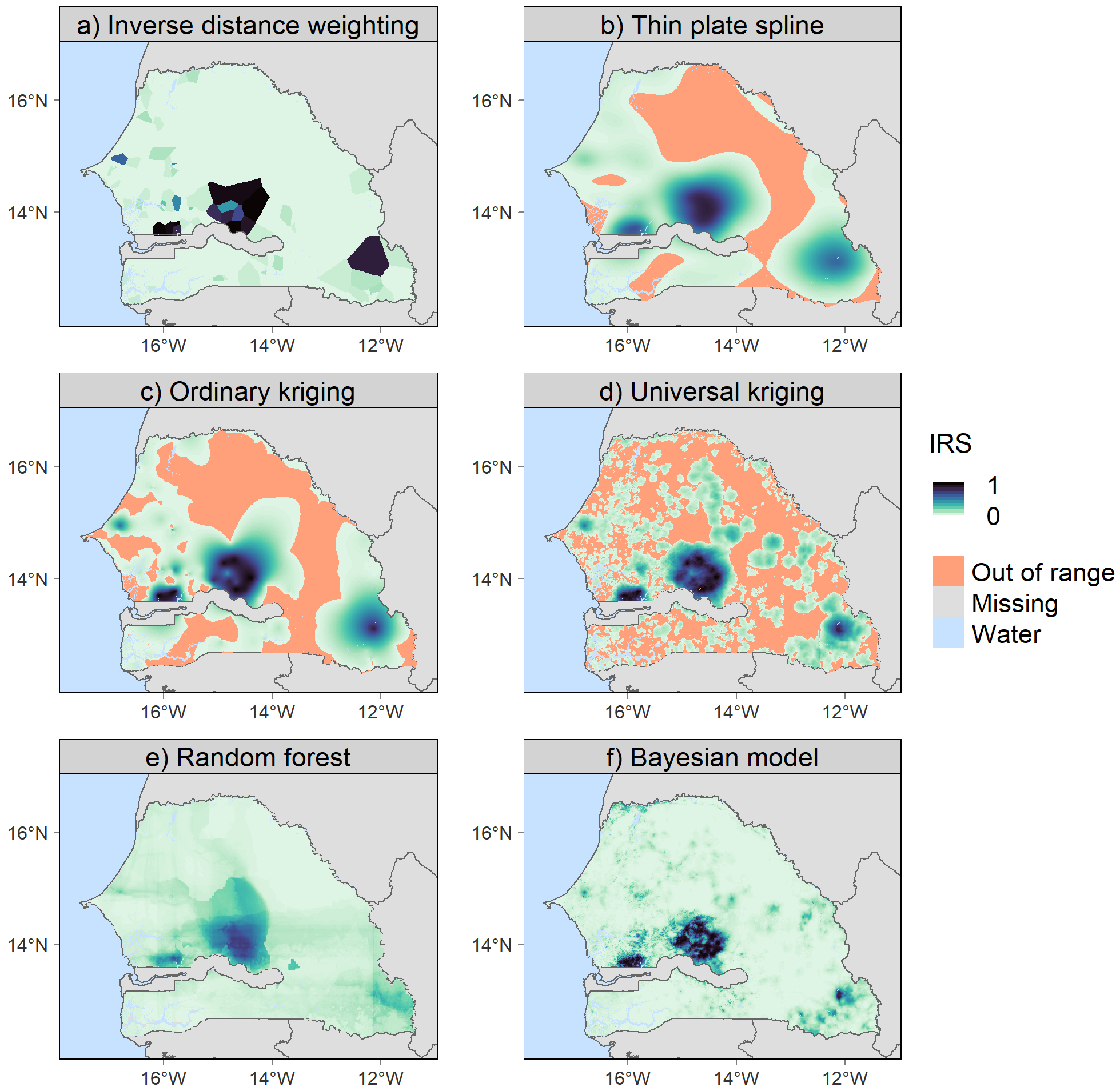

Supplement: S15 Fig — The maps show the spatial distribution of the proportion (ranging from 0 to 1) of households that were sprayed with a residual insecticide in the last year prior to the survey. Gridded surfaces are produced at a resolution of 1x1 km for all methods examined in the study. The ‘Out of range’ label indicates predicted values that are outside the possible range of values of the indicator (below 0 or above 1). Out-of-range predictions were made by thin plate spline, ordinary kriging and universal kriging. IRS stands for indoor residual spraying. National boundaries were downloaded from GADM. (TIF) [file pone.0322819.s020.tif]

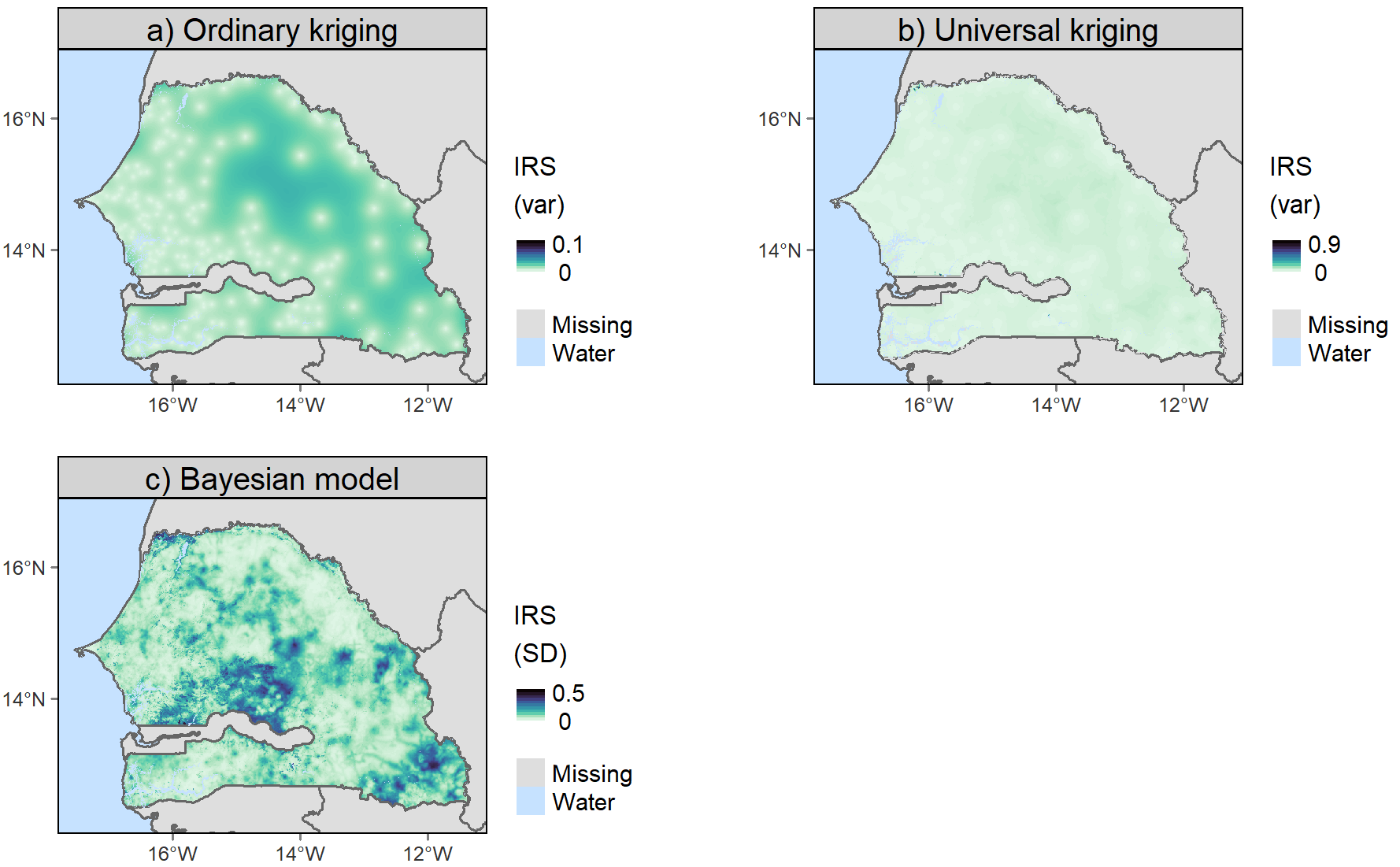

Supplement: S16 Fig — Uncertainty is measured as a prediction variance (var) for kriging methods (a, b) and as a standard deviation (SD) for Bayesian models (c). Higher values of SD or variance indicate areas with greater uncertainty in the predicted indicator, reflecting lower confidence in the accuracy of the predictions in these regions. IRS stands for indoor residual spraying. National boundaries were downloaded from GADM. (TIF) [file pone.0322819.s021.tif]

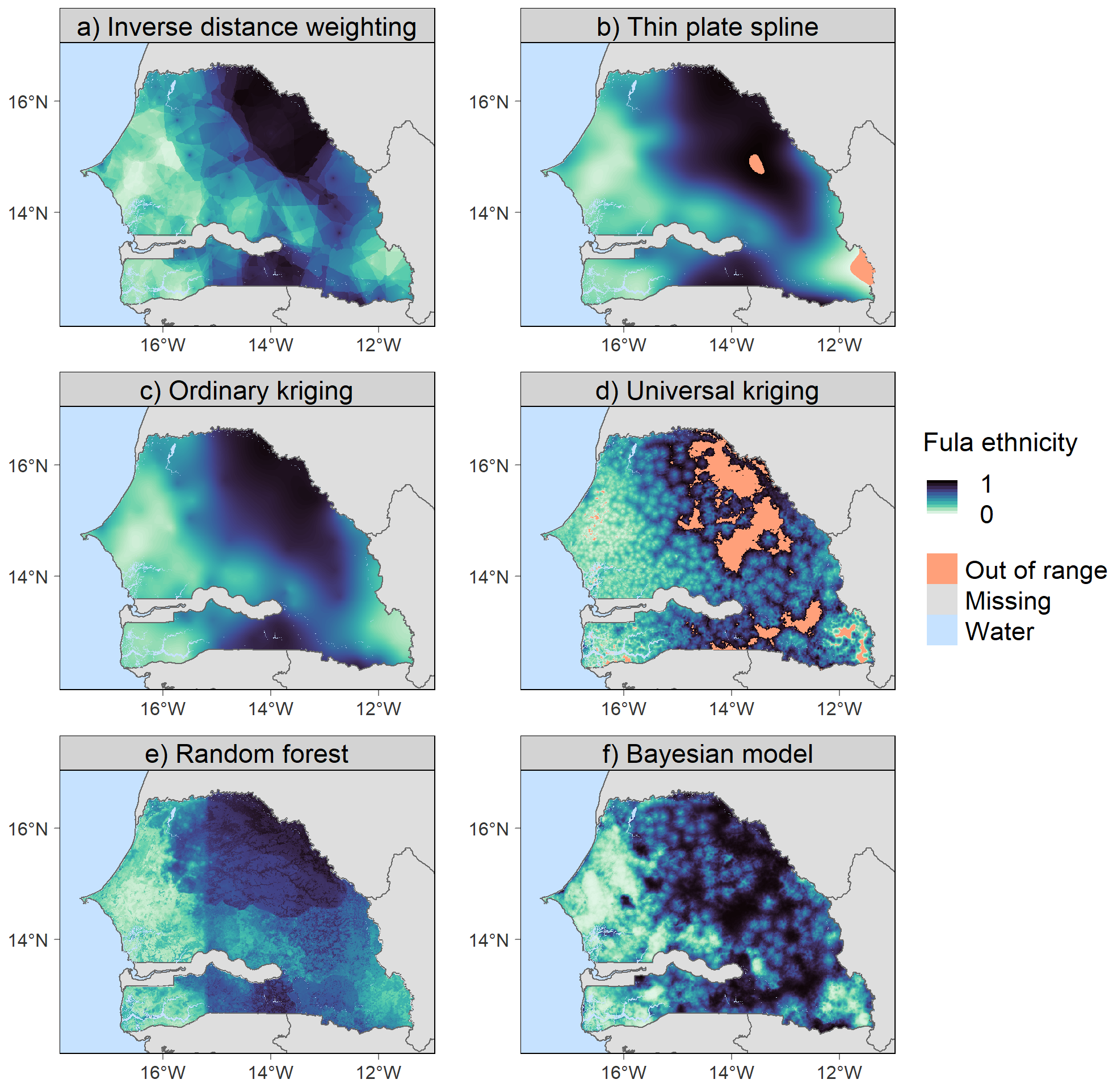

Supplement: S17 Fig — The maps show the spatial distribution of the proportion (ranging from 0 to 1) of people belonging to the Fula ethnic group in Senegal. Gridded surfaces are produced at a resolution of 1x1 km for all methods examined in the study. The ‘Out of range’ label indicates predicted values that are outside the possible range of values of the indicator (below 0 or above 1). Out-of-range predictions were made by thin plate spline and universal kriging. National boundaries were downloaded from GADM. (TIF) [file pone.0322819.s022.tif]

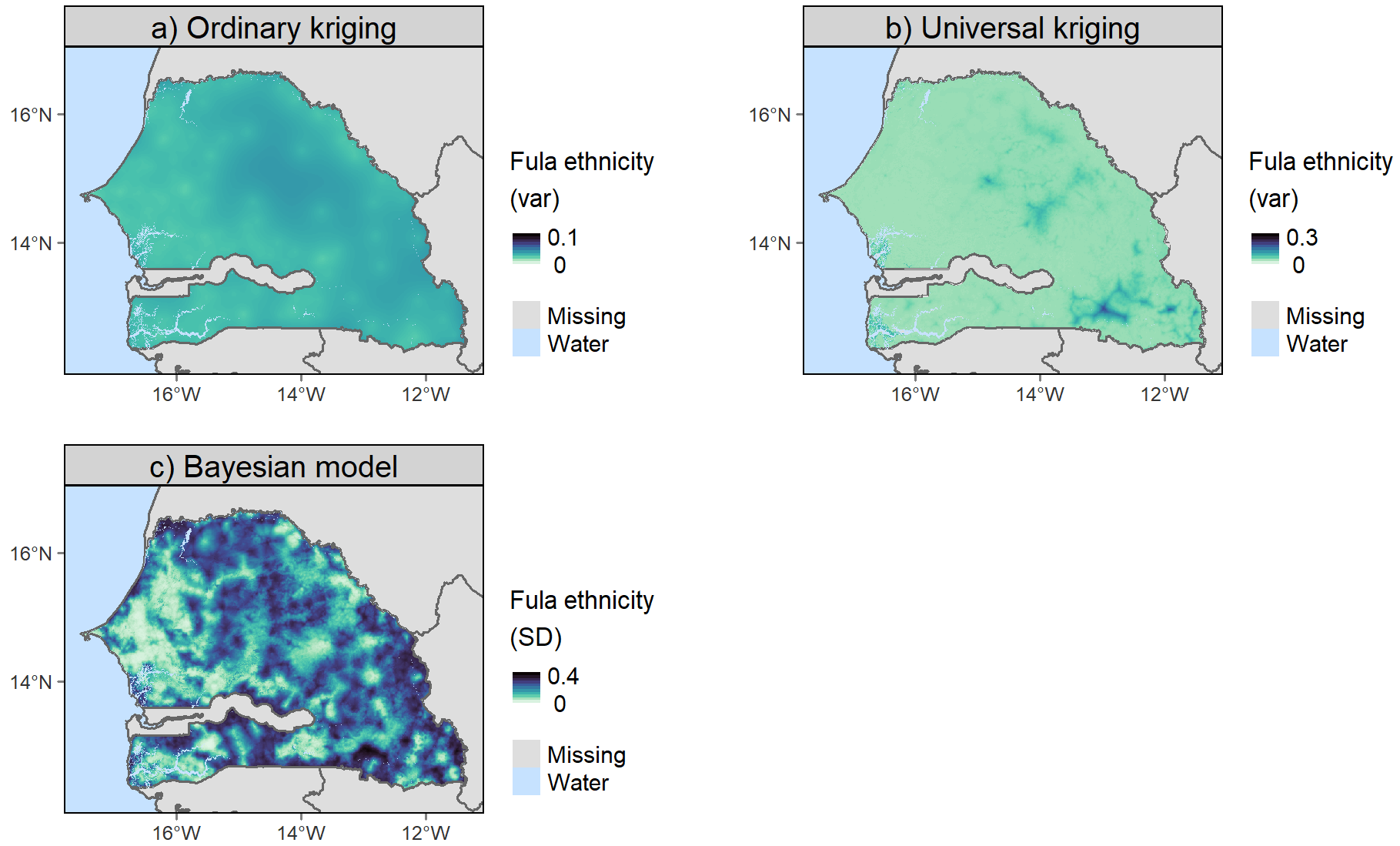

Supplement: S18 Fig — Uncertainty is measured as a prediction variance (var) for kriging methods (a, b) and as a standard deviation (SD) for Bayesian models (c). Higher values of SD or variance indicate areas with greater uncertainty in the predicted indicator, reflecting lower confidence in the accuracy of the predictions in these regions. National boundaries were downloaded from GADM. (TIF) [file pone.0322819.s023.tif]
